# Network architecture underlying maximal separation of neuronal representations

**DOI:** 10.3389/fneng.2012.00019

**Published:** 2013-01-03

**Authors:** Ron A. Jortner

**Affiliations:** Interdisciplinary Center for Neural Computation, Hebrew UniversityJerusalem, Israel

**Keywords:** neural coding, sparseness, circuit, connectivity, specificity, olfaction, insect, locust

## Abstract

One of the most basic and general tasks faced by all nervous systems is extracting relevant information from the organism's surrounding world. While physical signals available to sensory systems are often continuous, variable, overlapping, and noisy, high-level neuronal representations used for decision-making tend to be discrete, specific, invariant, and highly separable. This study addresses the question of how neuronal specificity is generated. Inspired by experimental findings on network architecture in the olfactory system of the locust, I construct a highly simplified theoretical framework which allows for analytic solution of its key properties. For generalized feed-forward systems, I show that an intermediate range of connectivity values between source- and target-populations leads to a combinatorial explosion of wiring possibilities, resulting in input spaces which are, by their very nature, exquisitely sparsely populated. In particular, connection probability ½, as found in the locust antennal-lobe–mushroom-body circuit, serves to maximize separation of neuronal representations across the target Kenyon cells (KCs), and explains their specific and reliable responses. This analysis yields a function expressing response specificity in terms of lower network parameters; together with appropriate gain control this leads to a simple neuronal algorithm for generating arbitrarily sparse and selective codes and linking network architecture and neural coding. I suggest a straightforward way to construct ecologically meaningful representations from this code.

## Introduction

Animals all use information about their surrounding world in order to function within it. Nervous systems have specialized in gathering, processing, storing, and retrieving such information and in using it to make decisions necessary for survival. To accomplish these tasks, the brain must disregard much of the information made available by the senses, extracting only what is relevant for the animal's needs. Just as in drawing a map of a newly discovered land, the brain, in so doing, creates a schematic internal representation of the animal's world—and it is over this internal model that generalizations are drawn, categories are discerned, associations made, and behavior triggered [Marr, 1970, 1971; Barlow, 1985; von der Malsburg, 1986, 1990; Kanerva, 1988; Földiák, 1990; reviewed in deCharms and Zador (2000)].

By virtue of the choice of what to keep in it, this internal neuronal representation is tailored to the organism's needs; and just as a historian, geologist, and meteorologist would each draw a different map of the same piece of land, it too suggests alternate ways of viewing and interpreting reality (Barlow, 1972; Kanerva, 1988; Churchland and Sejnowski, 1990). In other words, a subjective internal model of the world serves as a substrate for performing computations which—by predicting the outcome of actions in the real world—allow efficient decision-making, even in novel situations (von der Malsburg, 1986, 1990; Kanerva, 1988). This may be the core of what the brain does.

Olfactory systems, which are in evolutionary terms ancient and found even in simple animals, accomplish this task very efficiently. The signals they analyze are plumes of airborne molecules and complex mixtures thereof—variable signals occurring on highly noisy background (Kadohisa and Wilson, 2006; Raman and Stopfer, 2010; Raman et al., 2011)—and from this input they extract meaning (such as “food,” “predator,” or “potential sexual partner”), which is translated into behavioral output (actions such as foraging, escape, or courtship, respectively).

How is this task accomplished by neural hardware? Circuit architecture is a key to understand brain dynamics and function. A full characterization of neural circuitry—including cell types and their integrative properties (input–output functions), connectivity between them (statistics, pattern, signs, and strengths) and external input driving the network (rates, auto- and cross-correlations, synchrony, etc.)—is necessary, though not sufficient, for transcending the descriptive level and distilling the system's design principles (Churchland and Sejnowski, 1992). This in turn yields a deeper understanding of how basic network features and their interrelations give rise to its higher properties. Few biological neural systems, however, are presently characterized in sufficient detail; most are riddled with complexity, knowledge gaps, and high-dimensional parameter-spaces.

**Figure 1:**
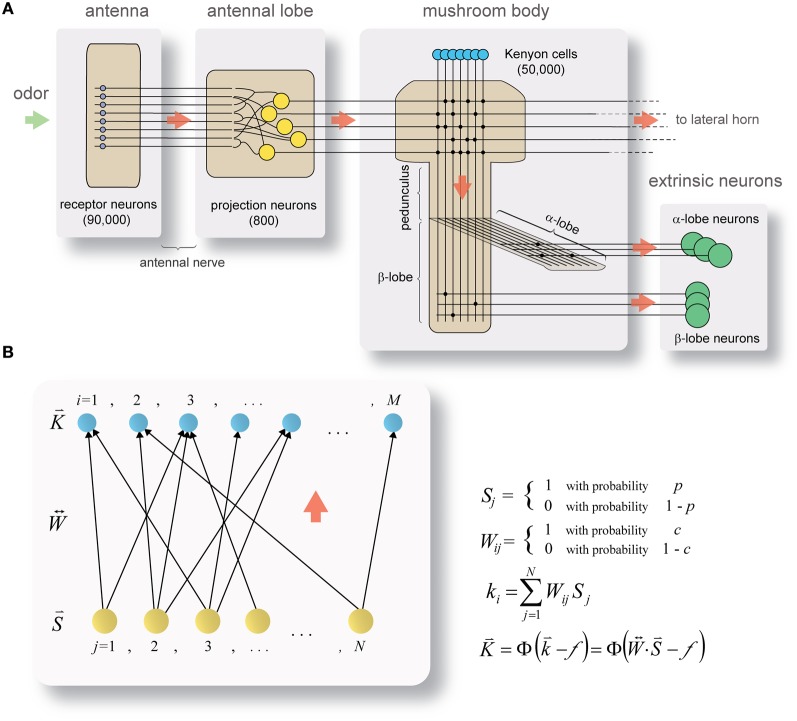
**Framework for studying the separation of neuronal representations. (A)** Circuit diagram of the locust olfactory system. Odor information reaches the antennal-lobe via ~90,000 olfactory receptor-neurons in the antenna. In the antennal-lobe, ~800 projection neurons (PNs, yellow) project it further to the mushroom body (onto ~50,000 Kenyon cells (KCs), blue; PN–KC connection probability ½) and the lateral horn (not shown). In transition from PNs to KCs the odor code dramatically changes from broad and highly distributed (in PNs) to sparse and specific (in KCs). KC axons split into the α- and β-lobes, where they synapse onto α- and β-lobe extrinsic neurons (green), respectively (KC–β-lobe-neuron connection probability ~0.02). Red arrows indicate direction of information flow. See text for more details. **(B)** Mathematical framework for studying transformation in coding. Model represents the state of a theoretical network inspired by PN–KC circuitry during a brief snapshot in time. Color code and information flow same as in **(A)**. A set of *N* source-neurons (activity of which is denoted by binary-valued vector $\vec{S}$; i.i.d. with probability *p*) projects onto a set of *M* target neurons (activity of which is denoted by vector $\vec{K}$) via a set of feed-forward connections (binary-valued connectivity matrix $\overset{↔}{W}$; i.i.d. with probability *c*). The aggregate input to the target layer $\vec{K}$ is the vector $\vec{K}$, a product of source-neuron activity vector $\vec{S}$ and connectivity matrix $\overset{↔}{W}$. $\vec{K}$ is obtained by thresholding $\vec{K}$ using the Heaviside function $\Phi (\vec{k}-f)$.

One example where detailed knowledge exists on network parameters and coding schemes is the olfactory system of the locust (*Schistocerca americana*) (Figure 1A). In this relatively simple system, 800 broadly tuned and noisy second-order neurons (projection neurons, PNs) project directly onto 50,000 third-order neurons (Kenyon cells, KCs), which are highly selective and reliable in their odor responses (Perez-Orive et al., 2002). As the system is feed-forward, small, well-defined, and displays a dramatic change in coding—from distributed to sparse—between source- and target-populations, it seems well suited for studying the origins of neuronal specificity.

The locust olfactory system (Figure 1A) receives odor input through the antenna, via ~90,000 olfactory receptor-neurons (ORNs) which terminate in the antennal-lobe. The antennal-lobe is a small network: ~800 excitatory PNs which send their axons to the next relays in the system (forming the antennal-lobe's sole output), and ~300 inhibitory interneurons (not shown in the diagram) which act locally within the network (Laurent and Davidowitz, 1994; Leitch and Laurent, 1996). PNs each respond to a wide array of odors with rich, complex spike-trains encoding odor identity (Laurent and Davidowitz, 1994; Laurent, 1996; Wehr and Laurent, 1996; Perez-Orive et al., 2002; Mazor and Laurent, 2005) and concentration (Stopfer et al., 2003); PN-spike-trains are additionally locked to a 20 Hz oscillatory cycle which is synchronous across the PN population (Laurent and Davidowitz, 1994; Laurent, 1996; Laurent et al., 1996) and is reflected in local-field-potential oscillations. With no odor presented, PNs fire spontaneously at rates of 2.5–4 Hz (Perez-Orive et al., 2002; Mazor and Laurent, 2005). Odors are represented by a dynamic combinatorial code (Laurent et al., 1996; Wehr and Laurent, 1996) which is broadly distributed across the PN population (Perez-Orive et al., 2002; Mazor and Laurent, 2005).

Output from the antennal-lobe is projected, via PN axons, onto two direct target-areas: the mushroom body, a structure involved in learning and memory (Heisenberg, 1998), and the lateral horn. The mushroom body contains ~50,000 small neurons, the KCs (Laurent and Naraghi, 1994; Leitch and Laurent, 1996). Individual KCs respond to specific odors (either monomolecular odors or mixtures), their responses are characterized by few spikes, are highly reliable across different presentations of the same odor (Perez-Orive et al., 2002), and are often concentration invariant (Stopfer et al., 2003). KC responses occur on a background of extremely little spontaneous firing (Laurent and Naraghi, 1994; Perez-Orive et al., 2002; Mazor and Laurent, 2005; Jortner et al., 2007; Jortner, 2009). Mushroom body odor-responses thus involve small, highly selective subsets of KCs (Perez-Orive et al., 2002, 2004; Stopfer et al., 2003; Jortner, 2009).

Axons of KCs exit the mushroom body calyx in a tight bundle (forming the mushroom's stalk, or pedunculus), branching into the mushroom body's output nodes, the α- and β-lobes (Laurent and Naraghi, 1994). There, KC output is integrated by smaller populations of extrinsic neurons (called α- and β-lobe neurons, respectively; Figure 1A) with large, planar dendritic trees which intersect KC-axon bundles at neat right angles (Li and Strausfeld, 1997; MacLeod et al., 1998; Cassenaer and Laurent, 2007), suggesting potential integration of precisely timed spikes over a wide KC-subpopulation.

Several previous studies offer theoretical treatment of the locust antennal-lobe–mushroom-body transformation (e.g., Garcia-Sanchez and Huerta, 2003; Theunissen, 2003; Huerta et al., 2004; Sivan and Kopell, 2004; Finelli et al., 2008); these, however, lack quantitative data regarding critical network parameters, such as connectivity values. More recent experimental work quantified aspects of network architecture via electrophysiological measurements of connectivity between PNs, KCs and β-lobe-neurons (Jortner et al., 2007; Cassenaer and Laurent, 2007). Results show that each KC receives synaptic connections from ½ of all PNs on average (~400 out of ~800 PNs); PN–KC synapses are very weak [excitatory-postsynaptic-potential (EPSP) amplitude is 85 ± 44 μV], and KC firing thresholds correspond to simultaneous activation of ~100 PN–KC synapses (assuming linear summation) (Jortner et al., 2007). Connections between KCs and some of their outputs (β-lobes neurons) are, on the other hand, sparse (~2% of pairs) and strong (EPSP amplitude 1.58 ± 1.1 mV), and exhibit Hebbian spike-timing-dependent plasticity (Cassenaer and Laurent, 2007).

Can these findings explain the transformation in coding schemes? What is the functional significance of this design? In the present study I explore design principles by which the brain constructs specific, sparse and high-level representations of the surrounding world. A coding strategy both sparse and selective would be one where *only a small subset of neurons respond to any given stimulus or external state* (i.e., high population sparseness; Willmore and Tolhurst, 2001), *and only a small subset of stimuli or external states elicit response in each neuron* (Jortner et al., 2007; Jortner, 2009). Inspired by the network architecture of the locust olfactory pathways, I suggest an exciting implementation of neuronal hardware to this end. My central claim is that in a feed-forward system with connectivity ½, target neurons differ maximally from each other in information they contain about the world (or external state); in this sense serving as an optimal neural module for parsing the world of inputs, and a substrate for sparse and specific neuronal-responses on the basis of which learning, categorization, generalization, and other essential computations can occur. The targets' sparseness is set to a controlled, arbitrary level by choice of a proper and adaptive firing threshold. Next, I address these points through a straightforward yet rigorous mathematical approach.

## Methods

The model I use is highly reduced, consisting of a layer of source-neurons (equivalent to PNs), projecting onto a layer of target neurons (equivalent to KCs) via a set of feed-forward connections (Figure 1B). Following several simplifying assumptions, I describe the mathematical framework and proceed to solve some of its behavior analytically—yielding predictions about function and about how network design relates to coding.

### Model assumptions

For the sake of tractability and predictive power, I make four important simplifying assumptions. First, I choose to look at a “snapshot” of the system in time; a brief-enough segment so that for any given PN the probability for spiking more than once is negligible. Within this time window, the PN population can be treated as a vector of binary digits, *one* denoting the occurrence of a spike and *zero* denoting none. As a second assumption, all PNs are treated each as firing (or not) within this time window with probability *p* which is identical across all PNs, and doing so independently of each other (i.i.d.); this allows treating the PN activity vector as binomial with a known parameter. Third, all synaptic connections are treated as equal in strength. As a fourth and last assumption, connectivity between PNs and KCs is assumed to be random, with i.i.d. statistics and probability *c* across all PN–KC pairs.

These assumptions, and particularly those of i.i.d. statistics of firing and connectivity, wield great predictive power; I will revisit them in the Discussion (Section “Regaining Complexity: Reexamining the Model's Initial Assumptions”), examine their validity with respect to experimental data on the locust olfactory system, and assess, wherever biological reality deviates from them (e.g., when some dependence and correlations are introduced), how model results may be affected.

### Model description

A schematic cartoon of the network-model appears in Figure 1B. There is a set of *N* source-neurons, denoted by vector $\vec{S}$ (so the neurons are *S*_1_, *S*_2_, …, *S*_*N*_): these are analogous to PNs in the antennal-lobe. A second set of *M* target neurons, denoted by vector $\vec{K}$ (so neurons *K*_1_, *K*_2_, …, *K*_*M*_), are analogous to KCs in the mushroom body. Source-neurons ($\vec{S}$) connect to target neurons ($\vec{K}$) through randomly determined connections of uniform strength; each PN can thus either connect to a given KC or not, with probabilities *c* and (1 − *c*), respectively. $\overset{↔}{W}$ is the connection matrix, with *W*_*ij*_ = 1 if the *j*th PN connects to the *i*th KC and 0 otherwise. Each row of $\overset{↔}{W}$ indicates the set of PNs physically connected to a given KC (so there are as many rows as KCs), and each column indicates the set of KCs receiving physical connections from a given PN (so there are as many columns as PNs). The rows I will refer to as the *connectivity vectors* to KCs.

As pointed out in the assumptions, the model looks at a snapshot of the neural system during a brief time window. Within it, each of the PNs can either fire a spike or not, and does so with probabilities *p* and (1 − *p*), respectively, so $\vec{S}$ also takes binary values. I call $\vec{S}$ the *activity vector* of the PN population, and S will be the set of all possible activity vectors, so $\vec{S}\in S$.

Formally, then (Figure 1B):

$$\begin{array}{l} S_{j}=\{\begin{array}{cc} 1 & \text{with probabil}.\\ 0 & \text{with probabil}. \end{array}\begin{array}{cc} & p\\ & 1-p \end{array}\;j=1,\;\ldots ,\;N\\ W_{ij}=\{\begin{array}{cc} 1 & \text{with probabil}.\\ 0 & \text{with probabil}. \end{array}\begin{array}{cc} & c\\ & 1-c \end{array}\;i=1,\;\ldots ,\;M;\;j=1,\;\ldots ,\;N \end{array}$$

During our given time window each of the *M* KCs receives PN inputs, which additively determine its “membrane-potential.” The input to each KC, to which I refer throughout this work as its *aggregate input* (denoted by *k*_i_ for the *i*th KC) is the sum of all PNs connected to it which fire during that time window, or formally

$$\begin{array}{l} \;\vec{k}=\overset{↔}{W}\;\cdot \;\vec{S}\\ k_{i}=\sum_{j=1}^{N}W_{ij}S_{j} \end{array}$$

Thus, $\vec{K}$ is a vector which takes natural values between 0 and *N* (according to how many of the PNs converging onto the KC fire). Each KC then fires a spike if and only if its aggregate input equals or exceeds the firing threshold, *f*, or

$$\begin{array}{l} \;\vec{K}=\Phi \left(\vec{k}-f\right)=\Phi \left(\overset{↔}{W}\;\cdot \;\vec{S}-f\right)\\ K_{i}=\Phi \left(k_{i}-f\right)=\Phi \left(\sum_{j=1}^{N}W_{ij}S_{j}-f\right) \end{array}$$

where Φ(*X*) denotes the Heaviside function:

$$\Phi (X)=\{\begin{array}{cc} 1 & \text{if }X\geq 0\\ 0 & \text{otherwise} \end{array}$$

So $\vec{K}$ is a binary-valued vector, *K*_*i*_ indicating whether or not the *i*th KC fires, and K is the set of all possible target-neuron activity vectors, so $\vec{K}\in K$. Thus, in this model, for a network with *N* PNs and *M* KCs (with threshold *f*) and a fixed connectivity matrix $\overset{↔}{W}$, a given state of the PN population (denoted by activity vector $\vec{S}$) unambiguously determines the activity vector of the KC population, $\vec{K}$.

### Mathematical conventions, symbols, and abbreviations

While the mathematics used throughout this work is mostly elementary, some of the derivations are nonetheless rather tedious. For the sake of clarity, they appear in shortened form within the text; I provide commented step-by-step derivations in the Appendix.

All but the most standard mathematical symbols used are defined the first time they appear. For quick reference, they are also listed in Table 1.

**Table 1:** **Mathematical symbols used throughout the paper**.

*c*	Probability of PN–KC connection (scalar, real within interval [0,1])
CDF	Cumulative Distribution Function
CLT	Central Limit Theorem
*D*(*x, y*)	Absolute difference between *x* and *y*, |*x* – *y*| (For binary values: (*x* – *y*)^2^)
*f*	Kenyon-cell firing threshold (in units of PN inputs)—(scalar, non-negative)
$H(\vec{X},\vec{Y})$	Hamming distance between binary-valued vectors $\vec{X},\vec{Y}$; number of bits by which they differ (scalar, real, non-negative)
i.i.d.	independent and identically distributed
$\vec{K}$	Activity vector of the Kenyon-cell population (vector, *Mx*1, binary values)
$\vec{K}$	vector of aggregate inputs to Kenyon cells (vector, *Mx*1, natural values)
K	Set of all possible Kenyon-cell activity vectors
*M*	Total number of Kenyon-cells (scalar, natural)
*N*	total number of PNs (scalar, natural)
*p*	PN-firing probability within characteristic time window (scalar, real within interval [0,1])
PDF	Probability Density Function
Pr(*x*)	Probability of *x*
*Q; Q(x)*	The Standard Normal cumulative distribution function; its value at *x*
$\vec{S}$	Activity vector of the PN population (vector, *Nx*1, binary values)
S	Set of all possible PN activity vectors
$\vec{U},\vec{V}$	Random PN–KC connectivity vectors; rows of $\overset{↔}{W}$ (vectors, 1 *x N*, binary values)
*u*,*v*	Random subsets of PNs
$\overset{↔}{W}$	Connectivity matrix between PNs and KCs (Matrix, *M x N*, Binary values)
Λ	variance of aggregate input to a KC (scalar, non-negative)
Φ (x)	The Heaviside (step) function; producing 1 if *x* ≥ 0 and 0 otherwise
Ψ	Mean aggregate input to a KC (scalar, real)
ρ (*x*,* y*)	Pearson's correlation coefficient between *x* and *y*
~	Equals in distribution
≡	Equals by definition
A∩B	Intersection of sets *A* and *B* (objects which belong to both *A* and *B*)
A∪B	Union of sets *A* and *B* (objects which belong to *A* or *B, inclusive or*)
*A* Δ *B*	Symmetric difference of sets *A* and *B* (objects belong to *A* or *B, but not both*)
‖ *A* ‖	Number of elements in set *A*
*A*\ *B*	Relative complement of sets *A* and *B* (objects belong to *A* and not to *B*)
*x*!	Factorial of *x*
|*x*|	Absolute value of *x*
〈*X*〉_*Y*_	Expected value of *X* over all possible values of *Y* (with their respective probabilities)
$\left[\begin{array}{l} x\\ y \end{array}\right]$	*x*-choose-*y*, the number of ways to pick *y* elements out of *x*

## Results

### Model results I: exploring properties of the connectivity matrix

Examining the set of connections between the neuronal populations $\vec{S}$ and $\vec{K}$ (connectivity matrix $\overset{↔}{W}$), we may ask to what extent two connectivity vectors (rows of $\overset{↔}{W}$) differ from each other. Let us calculate how many binary digits will, on average, differ across two such connectivity vectors (which I call $\vec{U}$ and $\vec{V}$). This difference-measure is the *Hamming distance* between the two vectors, denoted by $H(\vec{U},\vec{V})$. Since all elements of the connectivity matrix are independent from each other, we can simply calculate the probability that an element of $\vec{U}$ differs from the matching element in $\vec{V}$(detailed derivation in Appendix A1):

$$\begin{array}{l} \mathrm{Pr}(U_{j}\neq V_{j})=\mathrm{Pr}(U_{j}=1,\;V_{j}=0)+\mathrm{Pr}(U_{j}=0,\;V_{j}=1)\\ \;=c(1-c)+(1-c)c=2c(1-c) \end{array}$$

and multiply by the total number of elements *N* to get the Hamming distance:

$${\langle H(\vec{U},\;\vec{V})\rangle }_{\vec{U},\;\vec{V}}=N\;\cdot \;\mathrm{Pr}(U_{j}\neq V_{j})=2Nc(1-c)$$

**Figure 2:**
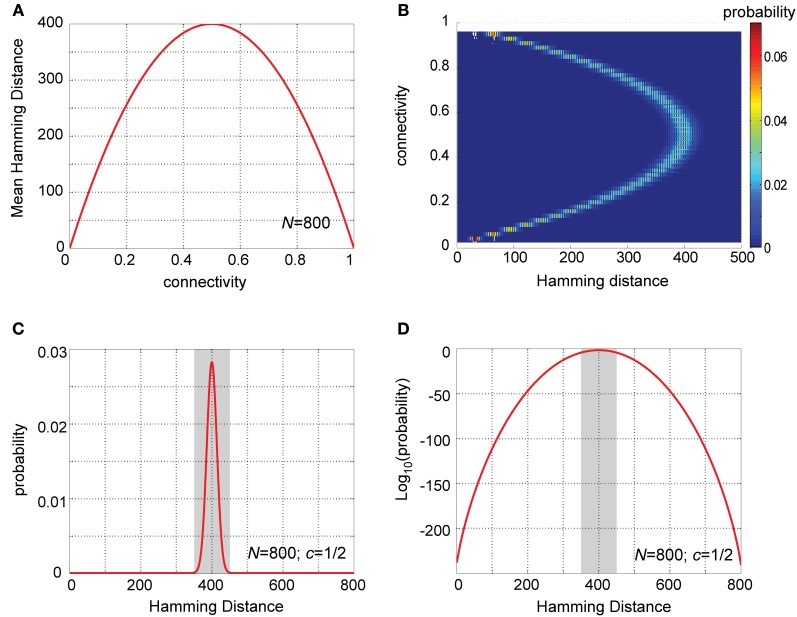
**Analysis of Hamming distances between PN–KC connectivity vectors. (A)** Analytic solution for mean Hamming distance between PN–KC connectivity vectors as a function of connectivity, *c* (for *N* = 800 PNs). Hamming distance is a parabola in *c* with maximum at *c* = ½. **(B)** Probability-density functions (PDFs) of Hamming distances between PN–KC connectivity vectors, Pr(*H*(*U*, *V*) = *j*), calculated as a function of connectivity. Each row corresponds to one PDF (with PN–KC connectivity value on ordinate); color represents probability. *N* = 800 PNs assumed in all cases. Note narrow distribution of Hamming distances around their mean for all values of *c*. Note PDF centered around highest value (400) is for *c* = ½. **(C)** Theoretically calculated PDF of Hamming distances between PN–KC connectivity vectors for parameters measured in the locust olfactory system (*N* = 800, *c* = ½). Note most common Hamming distance value, 400 (as each of two KCs samples on average 200 PNs that the other does not, see text). Shaded area, interval [350,450] in which most probability-density (0.9997) is concentrated. **(D)** Linear-log plot of same PDF as in **(C)**. Shaded area, interval [350,450]. Note miniscule values away from the mean (outside shaded area).

As this expression shows, when viewed as a function of the connection probability, *c*, the Hamming distance between two rows of $\overset{↔}{W}$ is maximal for *c* = ½, and drops symmetrically around it (Figure 2A, for *N* = 800). Thus, under the model assumptions, PN–KC connectivity vectors will on average be maximally different (as measured by Hamming distance) from each other when each pair of cells (PN and KC) is equally likely to be connected or not. This already suggests some special property of the experimentally observed connectivity matrix (Jortner et al., 2007).

If we now pick two connectivity vectors at random, what is the probability that they are identical? In other words, what is the probability that two randomly chosen KCs sample the exact same ensemble of PNs?

$$\begin{array}{l} \mathrm{Pr}(H\left(\vec{U},\;\vec{V}\right)=0)=\mathrm{Pr}\left(\sum_{j=1}^{N}{\left(U_{j}-V_{j}\right)}^{2}=0\right)\\ \;=\mathrm{Pr}\left(U_{j}=V_{j}|\forall j\right)\\ \;={\left(\mathrm{Pr}\left(U_{j}=1,\;V_{j}=1\right)+\mathrm{Pr}\left(U_{j}=0,\;V_{j}=0\right)\right)}^{N}\\ \;={\left(c^{2}+{\left(1-c\right)}^{2}\right)}^{N}={\left(2c^{2}-2c+1\right)}^{N} \end{array}$$

Similarly, the probability that the two connectivity vectors differ from each other by exactly *d* PNs is:

$$\begin{array}{l} \mathrm{Pr}(H(\vec{U},\;\vec{V})=d)=\mathrm{Pr}\left(\sum_{j=1}^{N}\text{​}{\left(U_{j}-V_{j}\right)}^{2}=d\right)=(\mathrm{Pr}\left(U_{j}=1,\;V_{j}=1\right)+\ldots \\ {\;+\mathrm{Pr}\left(U_{j}=0,\;V_{j}=0\right))}^{N-d}\;\cdot \;(\mathrm{Pr}\left(U_{j}=1,\;V_{j}=0\right)+\ldots \\ \;{+\mathrm{Pr}\left(U_{j}=0,\;V_{j}=1\right))}^{d}\;\cdot \;\left[\begin{array}{l} N\\ d \end{array}\right]\\ \;={\left(2c^{2}-2c+1\right)}^{N-d}\;\cdot \;{\left(2c(1-c)\right)}^{d}\;\cdot \;\frac{N!}{d!(N-d)!} \end{array}$$

This yields a theoretical probability-density function (PDF) for the Hamming distance between connectivity vectors (Figures 2B–D). Note that for all values of *c* the PDFs are always rather narrow (Figure 2B), with most of their mass concentrated close to their mean value. This is a key property of binomial distributions with large values of *N*, and implies that most pairs of connectivity vectors in a system obeying our basic assumptions will differ by similar values, well predicted by their mean Hamming distance. Note also, that the PDF centered on the highest value is for *c =* ½, the connectivity value measured between PNs and KCs in the locust. Figures 2C,D provide a closer look at this particular case (see next section).

**Figure 3:**
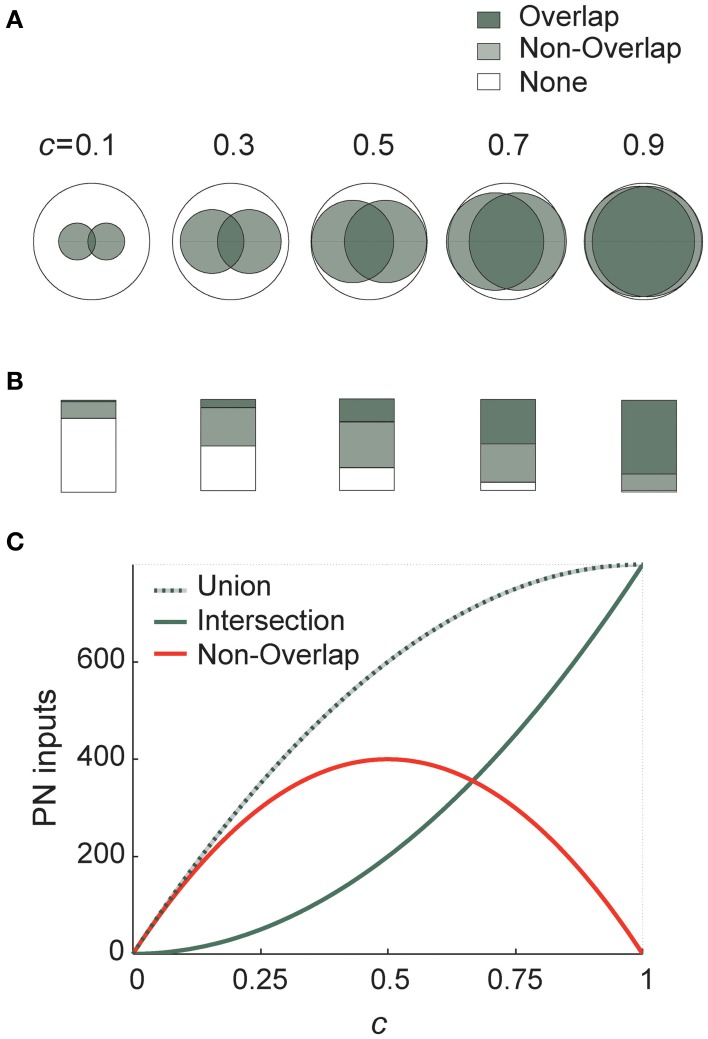
**Connection probability ½ maximizes differences between KC input-populations. (A)** Schematic representation of two KC inputs using Venn diagrams. Each large (empty) circle represents the entire PN population; the shaded circles within it represent two average KCs receiving connections from subsets of these PNs (with probability indicated above each diagram). Total shaded area (light-shaded + dark-shaded) represents the union of the two KC input-ensembles (or “receptive fields”), while the dark-shaded area alone is their intersection. The light gray area thus corresponds to the non-overlapping portion of the input-ensembles (union minus intersection), or to how different KCs are from each other in terms of input. **(B)** Same as in **(A)** using bar graphs. Each large rectangle represents the entire PN population; shaded areas use same color code and same connection probabilities as in **(A)**. **(C)** Analytically calculated curves of the union (dotted line), intersection (solid gray) and their difference (red) for two KCs in terms of PN input, as a function of PN–KC connection probability *c*. While the two former are both monotonically increasing, the latter is maximized at *c* = ½. Representations of the outside world are thus spread maximally across the target neuron population for connectivity ½.

Connectivity ½ thus maximizes differences between PN–KC connectivity vectors. I demonstrate this graphically in Figure 3 using elementary Venn diagrams. Two different KCs, each of which samples PNs randomly and independently with probability *c*, thus define two sets of PNs (I call these sets *u* and *v*). Each large (open) circle in Figure 3A represents the entire PN set (with area *N*), the two smaller circles within it mark the PN subsets *u* and *v* sampled by our two KCs (with average area *N* · *c* each; the value of *c* is indicated above each diagram).

The set of PNs sampled by both KCs (the *overlap* of the two PN sets) is the intersection of *u* and *v*, the number of PNs it includes on average is

$${\langle \left\|u\cap v\right\|\rangle }_{u,\;v}={\langle \sum_{j=1}^{N}U_{j}V_{j}\rangle }_{\vec{U},\;\vec{V}}=Nc^{2}$$

as demonstrated by the dark-shaded areas in Figures 3A,B. Similarly, the set of PNs sampled by *exactly* one of the two KCs (the *non-overlapping portion* of inputs to the two KCs, or their *symmetric difference*, Δ, in set theory terms) is the union of *u* and *v* minus their intersection; the average number of PNs it includes:

$$\begin{array}{l} {\langle \left\|u\Delta v\right\|\rangle }_{u,\;v}={\langle \left\|\left(u\cup v)\backslash (u\cap v\right)\right\|\rangle }_{u,\;v}\\ \;={\langle \sum_{j=1}^{N}U_{j}+\sum_{j=1}^{N}V_{j}-2\sum_{j=1}^{N}U_{j}V_{j}\rangle }_{\vec{U},\;\vec{V}}=2Nc(1-c) \end{array}$$

which corresponds to the light-shaded area in Figures 3A,B. This tells us how much these two KCs differ on average in PN ensembles they sample (or in their “receptive fields” in terms of input). This area is small when *c* is very low or very high, and maximal when *c =* 0.5 (as seen in Figure 3A, and more clearly in the bar graphs in Figure 3B). In fact, this expression is also precisely the result we got for Hamming distance between connectivity vectors (see above and Figure 2A). The white areas (“None” in Figures 3A,B) correspond to PNs not sampled by either of two KCs. Both the average union and average intersection of the two PN ensembles increase monotonically with connectivity, but the difference between them (the non-overlapping ensemble) peaks at ½ (Figure 3C).

Differences between receptive ranges (or “receptive fields”) of two target neurons are thus large when they each sample an *intermediate* proportion of the source-population—sampling either a very small or very large proportion yields much smaller non-overlapping ensembles, hence source-populations less different from each other.

### Properties of the connectivity matrix: plugging in real-data values

To sense how the above translates into biological reality, let us apply these calculations to the connectivity matrix of the locust olfactory system. For values relevant to the locust (*N* = 800 PNs and *c* = ½) the mean Hamming distance between two PN–KC connectivity vectors is 400; two randomly chosen KCs will thus overlap by 200 connected PNs on average, and each of the KCs will on average sample 200 PNs which the other does not. There will be an additional 200 PNs which are not sampled by either of the two KCs.

Figures 2C,D show the predicted distribution of Hamming distances between PN–KC connectivity vectors in the locust. Note the mean Hamming distance between two KC connectivity vectors (400) is also by far the most common value; it occurs with probability 0.028. The main mass of the distribution is tightly concentrated around the mean value (Figure 2C): 0.9997 out of a total mass of 1 of the PDF lies within ±50 PN inputs from the mean (shaded area in Figure 2C); randomly chosen pairs of KCs will thus almost always (in 99.97% of cases) have input-ensembles differing by 350–450 PNs. The PDFs take extremely small values further away from the mean, as better seen on a semi-logarithmic scale (Figure 2D, shaded area is same interval): note the miniscule probabilities outside the interval 350–450. The probability that two different KCs will sample the exact same PN ensemble is ~10^−241^, and the probabilities that their input-ensembles will differ by 1, or 2, or 3 inputs are 10^−238^, 10^−235^, and 10^−233^, respectively—vanishingly small numbers in all these cases.

### Model results II: neuronal activity and properties of input to KCs

**Figure 4:**
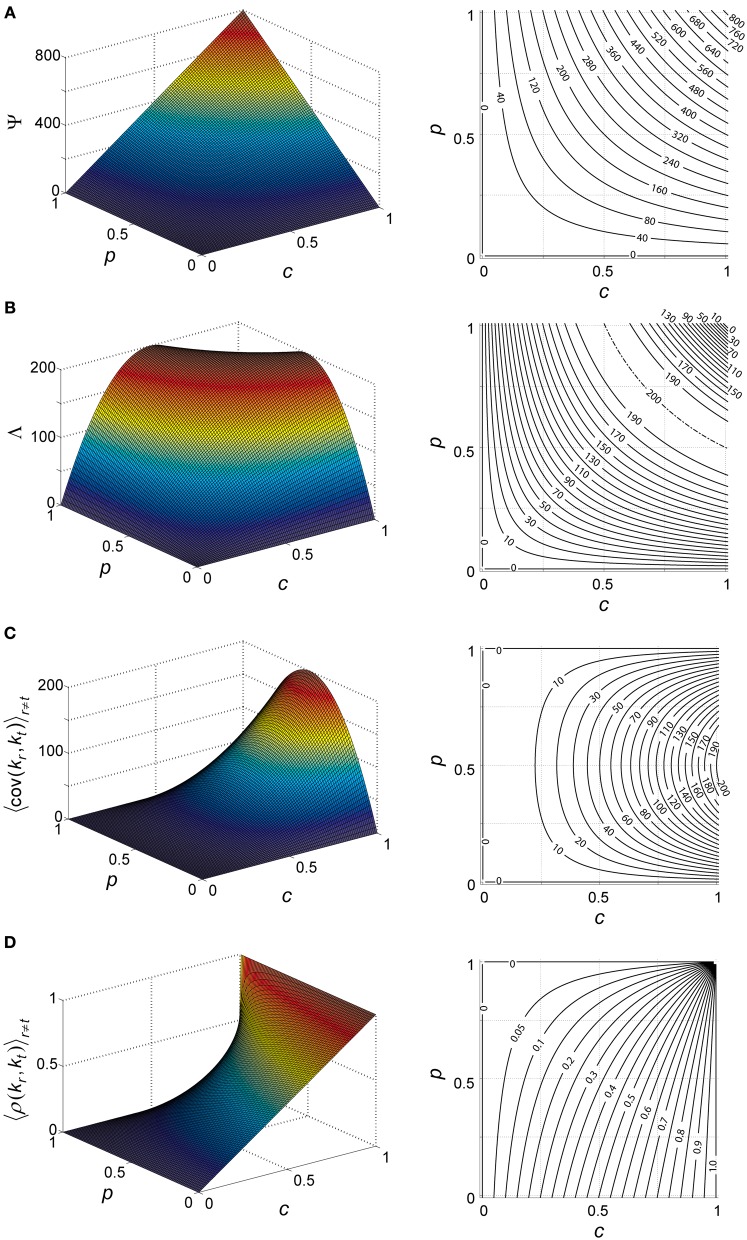
**Theoretical properties of network input to KCs. (A–D)** Analytically calculated properties of the aggregate input to KCs (*k*) during network activity. Aggregate input is also analogous to KC membrane-potential (see text). Each property plotted as a function of PN-spiking probability *p* and PN–KC connectivity *c*, and averaged over all antennal-lobe states. *N* = 800 PNs is assumed in all cases. Left, surface plot; right, contour plot. Contour intervals are identical within each plot. Dash-dot lines indicate ridge contours. For clarity, isoline values are sometimes indicated beside plot (when contour lines are too dense for inline labeling). **(A)** Mean aggregate input per KC, Ψ [units of PNs]; contour interval, 40. **(B)** Variance of aggregate input per KC, Λ [units of PNs]; contour interval, 10. **(C)** Covariance between aggregate inputs to two KC [units of PNs]; contour interval, 10. **(D)** Correlation coefficient between aggregate inputs to two KC [unitless]; contour interval, 0.05.

Up until now, we only considered the properties of the connectivity matrix, $\overset{↔}{W}$. To see what happens when neural activity is added in, let us put some flesh on the dry skeleton, and proceed to explore the aggregate input to KCs ($\vec{K}$) during network activity—corresponding to their sub-threshold membrane-potential. The symbol Ψ denotes the mean aggregate input to a KC, averaged over all possible PN-population states and across all KCs. Then

$$\begin{array}{l} \Psi \equiv {\langle k_{i}\rangle }_{\vec{S},\;i}={\langle {\langle \sum_{j=1}^{N}W_{ij}S_{j}\rangle }_{\vec{S}}\rangle }_{i}={\langle \sum_{j=1}^{N}W_{ij}{\langle S_{j}\rangle }_{\vec{S}}\rangle }_{i}\\ \;=p\;\cdot \;\sum_{j=1}^{N}{\langle W_{ij}\rangle }_{i}=Npc \end{array}$$

the mean aggregate input to a KC during our arbitrary time window is thus a simple product of the number of PNs, probability of spiking in a single PN during this snapshot and PN–KC connection probability (Figure 4A).

Λ will denote the variance of *k*_*i*_, averaged across all KCs and over all possible PN-population states (Figure 4B) (see Appendix A2 for full derivation):

$$\begin{array}{l} \Lambda \equiv {\langle \text{var}(k_{i})\rangle }_{i}={\langle {\langle {\left(k_{i}-{\langle k_{i}\rangle }_{\vec{S}}\right)}^{2}\rangle }_{\vec{S}}\rangle }_{i}\\ \;={\langle {\langle {\left(\sum_{j=1}^{N}W_{ij}S_{j}-\Psi \right)}^{2}\rangle }_{\vec{S}}\rangle }_{i}\\ \;=\sum_{j=1}^{N}\sum_{k=1,\;j\neq k}^{N}{\langle W_{ij}W_{ik}\rangle }_{i}{\langle S_{j}S_{k}\rangle }_{\vec{S}}+\ldots \\ \;+\sum_{j=1}^{N}{\langle W_{ij}^{2}\rangle }_{i}{\langle S_{j}^{2}\rangle }_{\vec{S}}-2\Psi \;\cdot \;\sum_{j=1}^{N}{\langle W_{ij}\rangle }_{i}{\langle S_{j}\rangle }_{\vec{S}}+\Psi^{2}\\ \;=Npc(1-pc) \end{array}$$

So we have explicitly expressed the mean and variance of the aggregate input *k*_*i*_ (Figures 4A,B) as a function of basic network parameters. Note that variable *k*_*i*_ is a product of two mutually independent, binomially distributed variables: the momentary vector of spiking in the PN population [a binomial with parameters (*N, p*)], and the vector of connections between the PN set and the KC [a binomial with parameters (*N, c*)]. Their dot product, *k*_*i*_, is also a binomial variable, with parameters *N* and *p* · *c*, as indicated by the calculations of Ψ and Λ.

To what extent are aggregate inputs to two KCs correlated with each other? Calculating their covariance (Figure 4C) we get (Appendix A3):

$$\begin{array}{l} {\langle \text{cov}(k_{r},\;k_{t})\rangle }_{r\neq t}={\langle {\langle \left(k_{r}-{\langle k_{r}\rangle }_{\vec{S}}\right)\left(k_{t}-{\langle k_{t}\rangle }_{\vec{S}}\right)\rangle }_{\vec{S}}\rangle }_{r\neq t}\\ \;={\langle {\langle \left(\sum_{i=1}^{N}W_{ri}S_{i}-\Psi \right)\left(\sum_{j=1}^{N}W_{tj}S_{j}-\Psi \right)\rangle }_{\vec{S}}\rangle }_{r\neq t}\\ \;=\sum_{i=1}^{N}\sum_{j=1,\;i\neq j}^{N}{\langle W_{ri}W_{tj}\rangle }_{r\neq t}{\langle S_{i}S_{j}\rangle }_{\vec{S}}+\ldots \\ \;+\sum_{i=1}^{N}{\langle W_{ri}W_{ti}\rangle }_{r\neq t}{\langle S_{i}^{2}\rangle }_{\vec{S}}-\ldots \\ \;-\Psi \sum_{i=1}^{N}{\langle W_{ri}\rangle }_{r\neq t}{\langle S_{i}\rangle }_{\vec{S}}-\ldots \\ \;-\Psi \sum_{j=1}^{N}{\langle W_{tj}\rangle }_{r\neq t}{\langle S_{j}\rangle }_{\vec{S}}+\Psi^{2}=Nc^{2}p(1-p) \end{array}$$

and their correlation coefficient (Figure 4D) is:

$$\begin{array}{l} {\langle \rho (k_{r},\;k_{t})\rangle }_{r\neq t}={\langle \frac{\text{cov}(k_{r},\;k_{t})}{\sqrt{\text{var}(k_{r})\;\cdot \text{ var}(k_{t})}}\rangle }_{r\neq t}=\frac{Nc^{2}p(1-p)}{\sqrt{{\left(Npc(1-pc)\right)}^{2}}}\\ \;=\frac{c-pc}{1-pc} \end{array}$$

Note that both covariance and correlation coefficient have non-negative values in our model (as *p* and *c* are probabilities, 1 ≥ *p*, *c* ≥ 0, and *N* is the number of PNs, *N* ≥ 0); this is expected in a network with architecture as described—with all connections feed-forward and excitatory—and with no correlations assumed between external inputs to the system. For *c* = 1, the correlation coefficient is 1 (as all KCs see the exact same input); for *c =* ½ the correlation coefficient is $\frac{1-p}{2-p}$, ranging between 0 and ½.

### Model results III: inter-KC difference is maximal for connectivity ½

We now touch a fundamental question: for given network parameters, how much do target neurons differ from each other in their aggregate inputs? This will tell us how much two KCs differ in sub-threshold membrane-potentials within a given cycle in the active network (earlier we asked how connectivity vectors differ; Section “Model Results I: Exploring Properties of the Connectivity Matrix”). Let us calculate the *difference, D*(*X*, *Y*) ≡ |*X* − *Y*|, between aggregate inputs to two KCs (see Appendix A4 for alternative derivation):

$$\begin{array}{l} D(k_{r},\;k_{t})\equiv {\langle {\langle \left|k_{r}-k_{t}\right|\rangle }_{\vec{S}}\rangle }_{r\neq t}=N\;\cdot \;{\langle {\langle {\left(W_{ri}S_{i}-W_{ti}S_{i}\right)}^{2}\rangle }_{\vec{S}}\rangle }_{r\neq t}\\ \;=N\;\cdot \;{\langle {\langle {\left(W_{ri}S_{i}\right)}^{2}-2W_{ri}W_{ti}S_{i}^{2}+{\left(W_{ti}S_{i}\right)}^{2}\rangle }_{\vec{S}}\rangle }_{r\neq t}\\ \;=N\;\cdot \;({\langle W_{ri}\rangle }_{r\neq t}\cdot {\langle S_{i}\rangle }_{\vec{S}}-2{\langle W_{ri}W_{ti}\rangle }_{r\neq t}\cdot {\langle S_{i}^{2}\rangle }_{\vec{S}}+\ldots \\ \;+{\langle W_{ti}\rangle }_{r\neq t}\cdot {\langle S_{i}\rangle }_{\vec{S}})\\ \;=N\;\cdot \;\left(cp-2c^{2}p+cp\right)=2pNc\left(1-c\right) \end{array}$$

**Figure 5:**
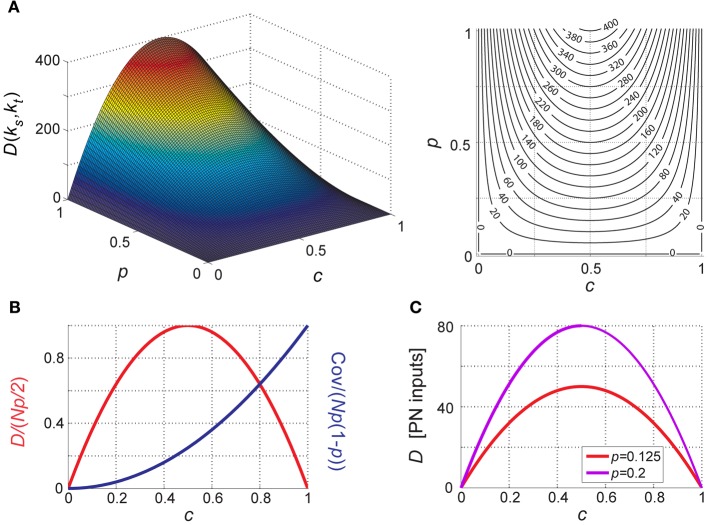
**Distance between KC inputs (or membrane-potentials). (A)** Difference (*D*) between aggregate inputs to two different KCs (averaged over all pairs of different KCs and over all antennal-lobe states) as a function of PN-firing probability *p* and PN–KC connection probability *c*. *D* is a parabola in *c* (with maximum at *c* = ½) and increases linearly with *p* and with *N*. *N* = 800 PNs is assumed. *D* is in PN inputs. Left, surface plot; right, contour plot, contour interval is 20. **(B)** Normalized difference and covariance between KCs as a function of connectivity *c*; normalized *D* and *cov* are unitless, independent of *N* and *p* and vary between 0 and 1. **(C)** Predicted difference between two KCs for PN-firing parameters measured of in the locust olfactory system (at both extremes of the range): *N* = 800, *p* = 0.125 (2.5 Hz, red), and *p* = 0.2 (4 Hz, magenta).

It is straightforward to see that when taken as a function of PN–KC connection probability, *D* is maximal for *c =* ½; this holds for any positive *p* and *N* (i.e., for all biologically relevant cases, with non-zero PN-firing probability and more than zero PNs in the network). The behavior of *D* as a function of *p* and *c* is shown in Figure 5A, and in normalized form in Figure 5B.

The above proves that when each target-cell samples half of the source-neurons, the mean difference between inputs to any two targets is maximized. Stated differently, each KC is on average maximally different from all other KCs in the information it carries about the external state.

### Inter-KC difference: plugging in real-data values

We can now introduce the values measured experimentally in the locust into our model. At baseline, PNs typically fire at ~2.5–4 Hz (Perez-Orive et al., 2002; Mazor and Laurent, 2005). The relevant integration time window for KCs is the 50 ms odor-induced oscillation cycle (Perez-Orive et al., 2002); even in the lack of oscillations EPSPs in KCs have a time course of several tens of milliseconds (Jortner et al., 2007). This provides a crude estimate of *p*, the probability of spiking within the relevant time window:

*p* ≈ 0.125–0.2 (Perez-Orive et al., 2002; Mazor and Laurent, 2005);
*c* ≈ 0.5 (PN–KC connectivity measurements; Jortner et al., 2007);
*N* ≈ 800 (axon count in the PN–KC tract; Leitch and Laurent, 1996).

Introducing these numbers into *D* = 2*pNc*(1 − *c*), the mean difference between two KCs is equivalent to 50–80 PN inputs (Figure 5C). If only 100 PNs converged onto each KC (*c* = 0.125), the mean difference would be 22–35 PNs, and with only 10 PNs per KC (*c* = 0.0125, as previously estimated; Perez-Orive et al., 2002), it would be equivalent to only 2.4–4 PNs at baseline (Figure 5C)!

During odor presentation, average PN firing-rates do not change significantly over the population (Mazor and Laurent, 2005). However, as PN-spikes are now confined to about half the oscillation cycle (the rising phase; Laurent and Davidowitz, 1994; Laurent et al., 1996; Wehr and Laurent, 1996), *p* effectively increases by ~factor 2 (by virtue of the time window “shrinking”). The mean difference between two KCs thus increases to 100–160 PNs during odor; if the fan-in were 100 PNs per KC (*c* = 0.125), or 10 PNs per KC (*c* = 0.0125), the mean difference would become 44–70 PNs, or 5–8 PNs, respectively.

### Model results IV: estimating firing threshold and sparseness

**Figure 6:**
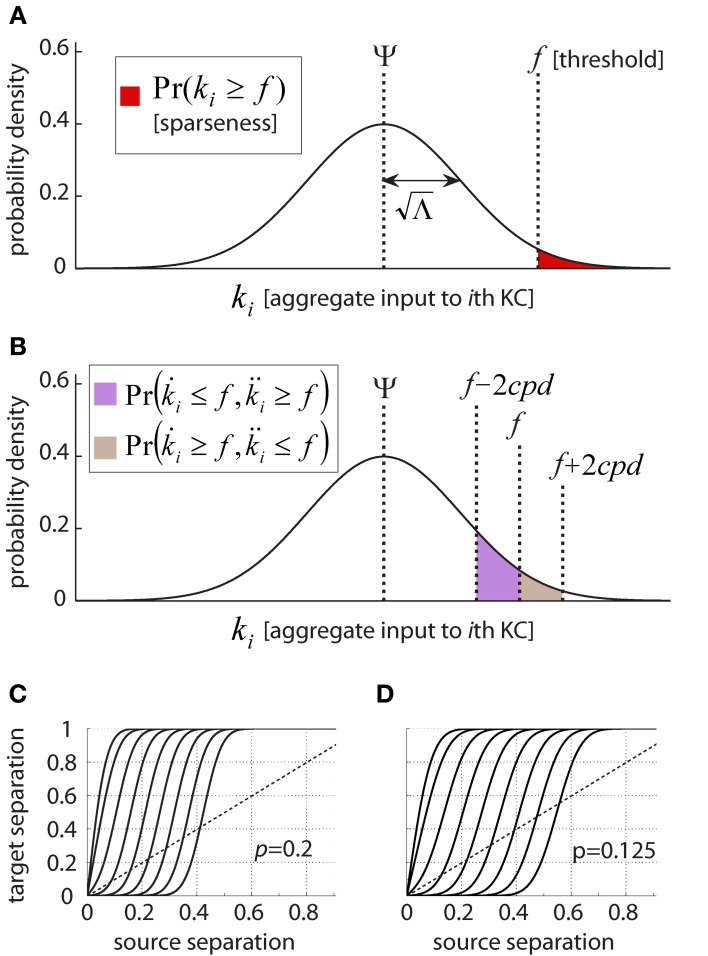
**Linking KC firing threshold and response properties. (A)** Dependence of Kenyon cell response sparseness on firing threshold. Kenyon cell aggregate input, or membrane-potential, *k*, behaves as a Gaussian random variable with mean Ψ and variance Λ under model assumptions; KC-response probability is the probability of aggregate input crossing firing threshold *f* (red shaded area). This defines both single-neuron selectivity (i.e., the average fraction of states which evoke firing in a given cell) as well as population sparseness (i.e., the fraction of KCs responding to an average state). **(B)** KC responses to partially overlapping PN activity patterns. Pairs of PN activity vectors differing from each other by exactly *d* bits present a KC with quantifiably different aggregate inputs. The summed probabilities of the KC responding to the first pattern and not the second (brown) or vice versa (violet) reflect the Hamming distance between KC activity vectors. This function thus expresses the difference between target-states given the difference between source-states. **(C–D)** Difference between Kenyon cell activity vectors as a function of difference between PN activity vectors. Difference is expressed as Hamming distance normalized by the vector-length (or number of neurons in the population). The nine different curves represent different KC firing thresholds, equivalent to (left to right) [0, 1, 2,…,8] standard deviations above the mean aggregate input. Above the diagonal (stippled line) are regimes where Kenyon cell activity vectors differ from each other more than the corresponding PN activity vectors: i.e., where pattern-separation occurs. Below the diagonal are regimes where different PN activity vectors evoke relatively close KC activity vectors: i.e., generalization/retrieval occurs. Parameters of the locust olfactory system are used, and **(C)** and **(D)** differ by PN-firing rate: **(C)**, *p* = 0.2, corresponding to PNs firing at 4Hz; **(D)**, *p* = 0.125, corresponding to PNs firing at 2.5 Hz; Note how increasing threshold affects curve shape and relative regimes of generalization vs. discrimination.

The above observations do not yet relate to KC response properties, as we up to now ignored membrane non-linearities and spiking. What happens when we impose a firing threshold, and assume the KC spikes once it is crossed? We now use the assumption of independence across PNs, and the fact that many of them respond to each odor and during each cycle (according to this model *N* · *p* per time window, or 100–160 PNs for values *N* = 800, *p* = 0.125–0.2. According to experimental data, 100–150 PNs fire per cycle; Mazor and Laurent, 2005). With these assumptions, we can apply the Central Limit Theorem (CLT) to the summation of inputs onto a KC: we can treat *k* as a Gaussian random variable, fully defined by its mean (Ψ) and variance (Λ) which we calculated (Section “Model Results II: Neuronal Activity and Properties of Input to KCs”):

$$\begin{array}{l} \;k_{i}=\sum_{j=1}^{N}W_{ij}S_{j}\\ k_{i}\sim \text{Norm}(\Psi ,\;\Lambda )=\text{Norm}(Npc,\;Npc(1-pc)) \end{array}$$

where Norm(*X, Y*) stands for a Normal distribution with mean *X* and variance *Y*. So for a given threshold *f* (in units of PN inputs), the probability of the *i*th KC crossing the threshold (i.e., spiking) is:

$$\begin{array}{l} \mathrm{Pr}(k_{i}\geq f)=\frac{1}{\sqrt{\Lambda 2\pi }}\int_{f}^{+\infty }e^{\frac{-{\left(x-\Psi \right)}^{2}}{2\Lambda }}dx\\ \;=1-\frac{1}{\sqrt{\Lambda 2\pi }}\int_{-\infty }^{f}e^{\frac{-{\left(x-\Psi \right)}^{2}}{2\Lambda }}dx=1-Q\left(\frac{f-\Psi }{\sqrt{\Lambda }}\right) \end{array}$$

where *Q*(*z*) denotes the Normal cumulative distribution function (CDF) of variable *z* (Figure 6A). If the firing threshold of KCs *f* is set to:

$$f=\Psi +z\;\cdot \;\sqrt{\Lambda }$$

this will result in a known and defined fraction of all KCs—equal to the area of the tail of the Gaussian [given by the CDF, 1 − *Q*(*z*)]—crossing the firing threshold for a given PN-population state. Similarly, a given KC will cross threshold *f* in response to a known fraction [again, the area of the tail 1 − *Q*(*z*)] of all PN-population states. This function, illustrated in Figure 6A, thus links the KC firing threshold with both the sparseness and the selectivity of the mushroom body neural code (as described in the Introduction).

To demonstrate the usage of this function: a hypothetical feed-forward network which satisfies our assumptions (Section “Model Assumptions”) has parameters *N* = 100, *p* = 0.5, and *c* = 0.2. If we wish to “design” a population of target neurons with a particular level of sparseness (say we want each responding to 2.3% of external states), we will set their firing thresholds to be equivalent to 2 standard deviations above their mean aggregate-input (as 1 − *Q*(2) = 0.023), or:

$$\begin{array}{l} f=Npc+2\;\cdot \;\sqrt{Npc(1-pc)}\\ \;=10+2\sqrt{9}=16\text{ simultaneous inputs}. \end{array}$$

A basic, gross prediction which naturally emerges from the threshold-sparseness function is that when the target neurons' threshold is equal to their mean aggregate input (Ψ), each target neuron responds to half of all external states [as *Q*(0) = 1 −*Q*(0) = 0.5]; this is the most-distributed code possible (also known as a holographic code; Földiák, 2002), and thus sparse coding is conditional on a threshold significantly higher than that, requiring *f* >> Ψ.

### Threshold estimation: plugging in real-data values

Let us now test the predictions on firing threshold and sparseness using experimental parameters from the locust. Section “Inter-KC Difference: Plugging in Real-Data Values” shows the network parameters for PN-firing rates (*p* ≈ 0.125–0.2), PN–KC connectivity (*c* ≈0.5) and PN number (*N* ≈ 800). Given these, the aggregate input a KC gets is on average

Ψ=Npc≈50−80 PN inputs per cycle

and its standard deviation is:

$$\sqrt{\Lambda }=\sqrt{Npc(1-pc)}\approx 7-8\text{ PN inputs per cycle}$$

So for KCs to each respond to ~1% of all PN-population states, their threshold has to be ~2.5 SDs above the mean aggregate input: *f* ≈ 98–101 PN-inputs for firing rate of 0.2 PN-spikes/cycle (4 Hz), or *f* ≈ 67–69 PN inputs for 0.125 PN-spikes per cycle (2.5 Hz).

This result is in good agreement with experimental measurements of KC-response sparseness (~1–2% using intracellular recordings; Jortner, 2009) and their firing threshold (*f* ≈ 100 assuming linear summation and full PN-synchrony; Jortner et al., 2007). The estimate's deviation toward the higher end of the predicted range may be due to supra-linear summation in KCs at depolarized membrane-potentials (Laurent and Naraghi, 1994; Perez-Orive et al., 2002, 2004).

### Model results V: noise tolerance and generalization

Olfactory stimuli are by nature noisy and variable. PNs show significant trial-to-trial variability when presented with the same odor repeatedly, yet in KCs noise is considerably reduced. Another issue is that some stimuli are similar to each other (either because of chemically related odorant molecules, or because they are mixtures with overlapping components) and others are different. Both points—the way the system tolerates noise and the way it encodes similar or different inputs—are closely related, in that both require us to examine how two overlapping PN-firing patterns are transformed into KC firing patterns.

Recall S, the set of all possible activity vectors of the source-population. Let us define $\left\{\vec{\dot{S}},\vec{\ddot{S}}\right\}$ as the subset of vector-pairs in S which differ from each other by exactly *d*-bits. Formally then:

$$\left\{\vec{\dot{S}},\;\vec{\ddot{S}}\in S|H(\vec{\dot{S}},\;\vec{\ddot{S}})=d\right\}$$

The aggregate-input vectors to the target-neuron population which are evoked by $\vec{\dot{S}},\vec{\ddot{S}}$ will be $\vec{\dot{k}},\vec{\ddot{k}}$, respectively; vectors $\vec{\dot{K}}$ and $\vec{\ddot{K}}$ will be the resulting activity vectors of the target-population. Aggregate inputs which a single KC gets in response to $\vec{\dot{S}},\vec{\ddot{S}}$ will differ by:

$$\begin{array}{l} {\langle D({\dot{k}}_{r},\;{\ddot{k}}_{r})\rangle }_{\left\{\vec{\dot{S}},\;\vec{\ddot{S}}\right\}}={\langle \left|{\dot{k}}_{r}-{\ddot{k}}_{r}\right|\rangle }_{r,\;\{\vec{\dot{S}},\;\vec{\ddot{S}}\}}\\ \;=N\;\cdot \;{\langle {\left(W_{rj}{\dot{S}}_{j}-W_{rj}{\ddot{S}}_{j}\right)}^{2}\rangle }_{r,\;\{\vec{\dot{S}},\;\vec{\ddot{S}}\}}\\ \;=N\;\cdot \;({\langle W_{rj}\rangle }_{r}\;\cdot \;{\langle {\dot{S}}_{j}\rangle }_{\left\{\vec{\dot{S}},\;\vec{\ddot{S}}\right\}}-2{\langle W_{rj}\rangle }_{r}\;\cdot \;{\langle {\dot{S}}_{j}{\ddot{S}}_{j}\rangle }_{\left\{\vec{\dot{S}},\;\vec{\ddot{S}}\right\}}+\ldots \\ \;+{\langle W_{rj}\rangle }_{r}\;\cdot \;{\langle S_{j}\rangle }_{\left\{\vec{\dot{S}},\;\vec{\ddot{S}}\right\}})\\ \;=Nc\;\cdot \;\left({\langle {\dot{S}}_{j}\rangle }_{\left\{\vec{\dot{S}},\;\vec{\ddot{S}}\right\}}-2{\langle {\dot{S}}_{j}{\ddot{S}}_{j}\rangle }_{\left\{\vec{\dot{S}},\;\vec{\ddot{S}}\right\}}+{\langle S_{j}\rangle }_{\left\{\vec{\dot{S}},\;\vec{\ddot{S}}\right\}}\right)\\ \;=Nc\left(p-2p\left(1-\frac{d}{N}\right)+p\right)=2cpd \end{array}$$

and so

$${\ddot{k}}_{r}={\dot{k}}_{r}\mp 2cpd$$

Earlier we linked KC aggregate input and firing threshold to their firing probability (Section “Model Results IV: Estimating Firing Threshold and Sparseness”; Figure 6A); let us use the same formalism now. The probability that a single KC responds differently to two PN-states is simply the probability one of these states drives it across the threshold and the other does not. This is demonstrated graphically in Figure 6B and is exactly the rationale behind the following calculation:

$$\begin{array}{l} {\langle D({\dot{K}}_{i},\;{\ddot{K}}_{i})\rangle }_{\left\{\vec{\dot{S}},\;\vec{\ddot{S}}\right\}}={\langle \left|{\dot{K}}_{i}-{\ddot{K}}_{i}\right|\rangle }_{\left\{\vec{\dot{S}},\;\vec{\ddot{S}}\right\}}\\ \;=\mathrm{Pr}\left({\dot{K}}_{i}=1,\;{\ddot{K}}_{i}=0\right)+\mathrm{Pr}\left({\dot{K}}_{i}=0,\;{\ddot{K}}_{i}=1\right)\\ \;=\mathrm{Pr}\left({\dot{k}}_{i}\geq f,\;{\ddot{k}}_{i}<f\right)+\mathrm{Pr}\left({\dot{k}}_{i}<f,\;{\ddot{k}}_{i}\geq f\right)\\ \;=\mathrm{Pr}\left({\dot{k}}_{i}\geq f,\;{\dot{k}}_{i}<f+2cpd\right)+\ldots \\ \;+\mathrm{Pr}\left({\dot{k}}_{i}<f,\;{\dot{k}}_{i}\geq f-2cpd\right)\\ \;=\frac{1}{\sqrt{\Lambda 2\pi }}\int_{f}^{f+2cpd}e^{\frac{-{\left(x-\Psi \right)}^{2}}{2\Lambda }}dx+\ldots \\ \;+\frac{1}{\sqrt{\Lambda 2\pi }}\int_{f-2cpd}^{f}e^{\frac{-{\left(x-\Psi \right)}^{2}}{2\Lambda }}dx\\ \;=Q\left(\frac{f\;+\;2cpd\;-\;\Psi }{\sqrt{\Lambda }}\right)-Q\left(\frac{f\;-\;2cpd\;-\;\Psi }{\sqrt{\Lambda }}\right) \end{array}$$

What about the activity vectors for the KC population, given similar PN input? The mean Hamming distance between two KC activity-patterns given Hamming distance *d* between the PN activity patterns will simply be the above expression multiplied by the number of KCs, *M*. We can thus write:

$$\langle H(\vec{\dot{K}},\;\vec{\ddot{K}})|H(\vec{\dot{S}},\;\vec{\ddot{S}})=d\rangle =M\left(Q\left(\frac{f\;+\;2cpd\;-\;\Psi }{\sqrt{\Lambda }}\right)-Q\left(\frac{f\;-\;2cpd\;-\;\Psi }{\sqrt{\Lambda }}\right)\right)$$

### Noise tolerance and generalization: plugging in real-data values

So how well does the locust olfactory system tolerate noise? The results are shown in Figures 6C,D, where I feed into the relation derived in the previous section the parameters from the locust circuitry. Two PN activity-patterns, differing by 0–800 bits (*x* axis, normalized to 0–1) evoke KC activity-patterns differing by 0–50,000 bits (*y* axis, normalized to 0–1). The diagonal (stippled lines) in both figures shows where the hypothetical curve would pass if normalized distance between representations would not change in transition from PNs to KCs. In fact, the relation has a sigmoid shape, meaning PN patterns close to each other become even closer in the KC population; whereas PN patterns which are different become more different (note that distances are normalized to the population size). The nine different sigmoid curves show the relation between input- and output-overlap when the firing threshold is varied (left to right: 0–8 standard deviations above the mean aggregate input). The setting of the firing threshold clearly controls the boundary between generalization and discrimination; a boundary which is surprisingly sharp.

In the locust olfactory system, the KC threshold is located ~2.5 SDs above the mean aggregate input (commensurate with a sparseness of ~1% as observed; see Section “Threshold Estimation: Plugging in Real-Data Values”). As seen in Figures 6C,D, for this value the Kenyon cell population generalizes (or, tolerates noise) for PN patterns which are within up to ~50–100 bits away from the PN–KC connectivity vectors, and discriminates for ones which are farther. This means, that with parameters from the locust, the boundary between discrimination and generalization lies in a biologically realistic regime for highly sparse coding (recall that for binary 800–dimensional vectors, over 99.9% of space is removed 350–400 bits from any given vector; Figure 2).

### Summary of model results and predictions

This analytic model produces several insights and predictions, applicable to both the locust olfactory circuitry as well as to feed-forward systems in general. As the model was designed with generality in mind, its results depend only minimally on particular parameter values. Here is a brief summary:

1. In a feed-forward system with random connectivity, pairs of connectivity vectors from source- to target-population have a maximal Hamming distance for connection probability ½.
2. Hamming distances between connectivity vectors are mostly very similar to each other and to their mean value; connectivity vectors significantly more similar to each other (or, more different from each other) will be extremely rare (negligible).
3. Differences in aggregate input (or sub-threshold membrane-potential) between target neurons are maximal for *c* = ½. In the locust antennal-lobe–mushroom-body circuit, where such connectivity is realized, pairs of KCs thus differ from each other by an equivalent of 50–80 PN-inputs during baseline, and of 100–160 PN-inputs when odor is presented. These differences decrease significantly when connectivity shifts away from ½ (in either direction): with connectivity of 10 PNs per KC (as previously estimated; Perez-Orive et al., 2002) differences between KCs would be equivalent to only 2.4–4 PN inputs (~factor 20 lower than for *c* = ½).
4. The standard deviation of sub-threshold membrane-potential in target neurons is maximal when the product of spiking probability in the source-neurons (*p*) and connectivity between the source- and target-populations (*c*) is ½. In locust KCs, the standard deviation of membrane-potential is predicted to be equivalent to the sum of 7–8 PN-inputs, or ~0.6–0.7 mV, in good agreement with experimental measurements.
5. The covariance of aggregate inputs to two different target neurons will be maximal for *p* = ½, and will increase as ~*c*^2^.
6. Both the covariance and correlation coefficient between target neurons are predicted to be always positive under the assumptions taken. This is intuitive, given that no correlations were assumed in the external input driving the system, and only feed-forward excitatory connections exist.
7. The correlation coefficient between target neuron membrane-potentials is expected to range within 0–0.5 for *c* = ½. Particularly, in the locust, where *c* = ½ and PN-spiking probability is 0.125–0.2 per cycle (2.5–4 Hz), correlation coefficients between KCs are predicted to be 0.4–0.5. This remains to be tested experimentally with dual-intracellular KC recordings. A related test—namely measurements of correlations between single-KC membrane-potentials and local-field-potentials—yielded correlation coefficients around 0.3 (Jortner et al., 2007).
8. The response probability (and sparseness) of target neurons in a feed-forward system with parameters *N, p, c* is determined by their firing threshold, and is well approximated by the area of a Gaussian tail. The threshold-sparseness function predicts the fraction of states a target cell responds to, and the fraction of target cells responding to any given state. It generates the basic prediction that for a threshold equivalent to the target neurons' mean aggregate input, Ψ (a product of source-neuron firing rate, source-neuron number and connectivity), target neurons will respond to ½ of all source-population states; so to produce sparse coding the threshold must exceed that value: *f* > > Ψ.
9. Applying the threshold-sparseness function to the locust olfactory system, the firing threshold measured (~100 inputs, assuming perfect synchrony and linear summation) well predicts the measured KC sparseness level (~1–2%) and vice-versa (1% sparseness predicts a threshold of ~70–100 inputs, depending on PN-firing rate).
10. Given a network with parameters *N*, *p*, and *c* = ½, if target neurons have a firing threshold of $f(z)=\frac{Np+z\cdot \sqrt{Np\left(2-p\right)}}{2}$ (see Appendix A5), then each target neuron will respond to 1 − *Q*(*z*) of source-population states, and different target neurons will respond to maximally different states. The difference between the target neurons will on average be $\frac{N\cdot p}{2}$. Combined with adaptive gain control to ensure that *f* is changed appropriately when *p* changes (Papadopoulou et al., 2011), this yields a simple way to design a network with an arbitrarily sparse level of activity, and with specific and reliable neural responses to external states.

## Discussion: linking network architecture and neural coding in the antennal-lobe–mushroom-body circuit

Integrating theory and experiment, I here discuss how the architecture of the locust olfactory system gives rise to Kenyon cell coding properties: specificity, reliability, low firing rates, correlations, and sparseness, and how these can be utilized to build higher-level representations of the animal's world. Several predictions with potentially broader implications will follow.

### Connectivity ½ maximizes differences between target neurons

The key experimental finding motivating this study was that each KC in the mushroom body receives synaptic connections from antennal-lobe PNs with probability ½, each thus sampling 400 of the 800 PNs (Jortner et al., 2007). At first, this result may seem very surprising—because it seems counterintuitive that KC specificity could arise from such broad PN inputs. It makes sense, however, when viewed from a combinatorics perspective: the number of ways to pick *n* elements out of *N* is given by the binomial coefficient:

$$\left[\begin{array}{l} N\\ n \end{array}\right]=\frac{N!}{n!(N-n)!}$$

This expression is maximal for *n = N/2*, decreasing sharply and symmetrically around it. The fundamental realization that choosing half the elements maximizes the number of possible combinations has dawned independently on several thinkers throughout history—from Pingala (India, 2nd–5th century BC, commentated by Halayudha, 10th century AD), through Al Karaji (Persia, 953–1029), Omar Khayyam (Persia, 1048–1131), Yang Hui (China, 1238–1298), Niccolo Tartaglia (Italy, 1500–1557) to Blaise Pascal (France, 1655).

How is this relevant to the olfactory system? Think of each KC as if picking the PNs it will listen to. If each KC sampled only *n* = 1 of *N* = 800 PNs, there would be exactly 800 ways to pick which PN to sample (similarly, if each KC sampled 799 out of the 800 PNs; where there would be exactly 800 ways to pick which PN *not* to sample). However, when sampling half the PNs, *n* = 400, the number of ways to do so is maximal, and equals 800!/400!400! ≈10^240^. This is an immense number—beyond astronomical—and way too large for any example from nature to demonstrate it. It is roughly equivalent to the number of atoms in the known universe (estimated at ~10^79^) taken to the power of three…

But as there are only 50,000 KCs in the locust mushroom body, only 5 · 10^4^ combinations are realized out of this vast pool of possibilities. What is the probability that two randomly chosen KCs sample the exact same PN-ensemble? The answer is ≈10^−240^, which is for all practical purposes zero. And what is the probability that two KCs sample very similar PN-ensembles—that is, ensembles differing from each other by just one, or 2 or 3 inputs? The answers are 10^−238^, 10^−235^, and 10^−233^, respectively—all vanishingly small. In fact, the average pair of KCs will differ by ~400 PN inputs (Figure 2C), which also constitutes the most common case (occurring with probability 0.028), and 99.97% of KC pairs will deviate from it by less than 50 inputs (Figures 2C,D). This stems from a key property of binomial distributions with large *N*: most of their mass occupies a very narrow band around their mean.

By this reasoning (proven for generalized cases in Sections “Model Results I: Exploring Properties of the Connectivity Matrix” and “Model Results III: Inter-KC Difference is Maximal for Connectivity ½” for connection vectors and membrane-potentials, respectively) each target neuron receives a unique set of source-neuron inputs, very different from that of all other target neurons. KCs are *maximally* different from each other in what they tell us about the world of inputs, because their connectivity vectors are drawn from a pool which is maximal. This feature of the KC population results directly from combinatorics, and from the probability ½ of receiving connections from their source-neurons (Figure 3).

A critical comment raised by several colleagues against the above argument is that while this architecture indeed maximizes input separation, this optimum cannot reflect on biological reality. The brain, they argue, could not come so close to it, because the numbers in question are too large to be distinguished from each other by a biological system. In other words, realizing 50,000 combinations of “only” 10^22^ (the number of ways to pick 10 PNs from 800, corresponding to connection probability *c* = 0.0125) would be already immensely sparse; and for all practical purposes 10^240^ (the number of ways to pick 400 from 800) is not “sparser.” Moreover, since the mathematical optimum is not necessary, evolution of such connectivity couldn't have possibly been guided by biological selection pressures.

The results presented here (Section “Inter-KC Difference: Plugging in Real-Data Values”; Figure 5) refute this criticism. While indeed the binomial coefficient $\left[\begin{array}{l} 800\\ m \end{array}\right]$ rises very steeply with *m* and soon produces vast numbers, these numbers directly translate into state-dependent differences in aggregate input, or membrane-potential, produced across KCs (Figure 5B, normalized difference; Figure 5C difference in inputs). If 80 PNs were to connect to each KC (corresponding to *c* = 0.1), the amount by which aggregate inputs to different KCs would differ—and the system's ability to discriminate between external states—would drop approximately to a third of the optimum, and for 10 PNs per KC (*c* = 0.0125) it would drop by a factor of 20. When translated to membrane-potential differences between KCs, this may be critical for readout, especially in the presence of noise. This maximum is thus likely to be meaningful after all, and may account for the exquisitely clean performance of sparse neural systems feeding on noisy input.

### What determines how sparse the code will be?

KC aggregate inputs differ maximally as a result of the PN–KC connectivity; yet while this property paves the road toward sparse coding, it does not in itself suffice to explain the KCs' rare firing: it is eventually their firing threshold which determines firing probability and response sparseness (Section “Model Results IV: Estimating Firing Threshold and Sparseness”). With such high convergence ratio (400:1), target cells can afford to have a very high firing threshold, which can account for KC specificity, reliability, and low firing rates. Experimental measurements show that KC firing threshold is equivalent to simultaneous activation of ~100 PN inputs assuming linear summation (Jortner et al., 2007). An estimate based on intracellular recordings (and thus less biased than extracellular studies, as it also captures cells firing rarely or not at all) suggests KCs respond to 1–2% of odors tested (Jortner, 2009). Here, a theoretical function was derived which links firing threshold and response probability (Section “Model Results IV: Estimating Firing Threshold and Sparseness”): it closely predicts the experimental results, estimating the firing threshold necessary to achieve ~1% KC sparseness at ~67–101 inputs, depending on PN-firing rates (Section “Threshold Estimation: Plugging in Real-data Values”).

The threshold-sparseness function is quite sensitive to activity levels of the input network (Huerta et al., 2004; Jortner et al., 2007; Nowotny, 2009). Since PN population activity produces a range (100–150) of spikes per cycle (Mazor and Laurent, 2005), this can result in instability of the code—causing some external states to activate a large number of KCs and others to activate none at all (Papadopoulou et al., 2011). This requires adaptive gain control of the KC firing threshold to fit the actual activity level of the input; one mechanism shown to maintain output sparseness over a wide range of input conditions in the locust takes place via a large, non-spiking GABAergic interneuron with extensive connectivity and graded release properties; it forms a negative-feedback loop onto KCs and adaptively regulates their population output on a cycle-to-cycle basis (Leitch and Laurent, 1996; Papadopoulou et al., 2011).

In a theoretical exploration of the hippocampus, O'Reilly and McClelland (1994) also find that a “floating threshold” (as they phrase it) is highly useful for determining response sparseness under different input conditions and postulate that adjustment of the threshold can be useful for shifting between pattern-separation (or discrimination, or new-category formation) and pattern-completion (or generalization, or recall).

It should be noted that the threshold-sparseness function derived here is independent from the results on connectivity, and it can be applied to systems with any parameters.

### Effects of PN–KC convergence: reliability and correlations

While overall PN–KC circuit-architecture is highly divergent due to the increase in dimensionality, the connectivity scheme makes single KCs receive massively convergent input (from 400 PNs). Together with the high and adaptive KC-threshold, this convergence sub-serves the KCs' reliable, low-noise performance: summing many PN-inputs prior to KC threshold (*f*) crossing is equivalent to massive averaging of PN activity. This reduces the significant variability (i.e., noise) present in individual, cycle-wise PN responses by a factor of $1/\sqrt{f}$; in locust KCs, where *f* ≈100, noise is thus reduced ~10-fold.

Another interesting effect of this convergence is the coexistence of correlations and differences in the mushroom body. While membrane potential-differences between different KCs are maximized, they are still predicted by the model to co-vary significantly (Figure 5B), with correlation coefficients of 0.4–0.5 [see Section “Summary of Model Results and Predictions”; Prediction (7)]. Indeed, while the mushroom body code is highly sparse and specific (Perez-Orive et al., 2002; Jortner, 2009), a salient property of KC intracellular membrane potentials is their strong correlations with the mushroom body local field potential (Laurent and Naraghi, 1994; Jortner et al., 2007), implying that they are also highly correlated with each other. How can strong correlations between cells, which we naturally tend to associate with similarity, exist side by side with maximal difference between them?

To answer this apparent paradox we examine the inputs KCs receive vs. the outputs they produce. Correlations between membrane-potentials of two KCs result from massive overlap in their aggregate input: they share on average ~200 incoming PN-synapses, and 25–40 active PN-inputs per oscillation cycle. The relevant feature for the system is, however, the number of inputs by which they do *not* overlap (Figure 3): each also receives on average ~200 PN synapses (25–40 active ones per cycle) which the other does not; so they differ by 400 synapses (50–80 active inputs per cycle). The non-linearity imposed by the KC-threshold makes the two properties—correlations and difference—strongly diverge at this point: two KCs can get highly correlated inputs, yet may easily sit across different sides of the threshold, which in turn determines who will fire and who won't; both correlations and differences can thus coexist between them.

The general message is that while sub-threshold correlations naturally arise from input overlap in highly interconnected systems, they do not necessarily imply similarity in function (or output) between neurons; depending on network design and on the parameter taken as readout, the non-overlapping input may outweigh the overlap (as shown for KCs). Eventually, the non-linearity of thresholding enables brains to parse the world into percepts and build representations from them. Membrane-potential correlations between KCs may in this case be side effects of the interconnected architecture, rather than a computational feature of the code.

### Neural design-principles for generating a sparse code

As pointed out in the Introduction, a prerequisite for understanding a neural system is characterizing its basic features—individual components, connectivity and external input. Formulating higher properties in terms of these features bridges levels of description and thus constitutes deeper understanding. This approach was used here link network design and sparse coding in the antennal-lobe–mushroom-body circuit. The experimentally measured parameters *f, c, p*—corresponding exactly to the individual unit input–output function, connectivity and input—were used to express distance-measures between connectivity vectors and between target neurons, sub-threshold behavior and coding sparseness.

Three main principles govern the design of the antennal-lobe–mushroom-body circuit: First, there are many more target cells than source cells (~50,000 vs. ~800); a factor ~10^2^ increase in dimensionality between the two odor-representations. Second, the probability of connection between the principal neurons of both relays is ~½; each target thus samples ~400 of 800 sources. Third, target-cell firing threshold is high, equivalent to simultaneously activating ~100 of their inputs, and can be fine-tuned to fit different activity-levels of the source network.

Due to the high threshold, each external state (or here, PN activity pattern) activates only a small subset of KCs. However, due to the connectivity scheme, different external states activate different KC-subsets. The activation of very small, very different subsets of cells in response to different external states suffices to produce a sparse and selective neural code as we defined it (see “Introduction”, and Jortner et al., 2007). At the same time, the high PN convergence onto individual KCs explains why KCs are so reliable on the one hand, and on the other hand why their membrane-potentials are noticeably correlated with the local-field potential (Laurent and Naraghi, 1994; Perez-Orive et al., 2002; Jortner et al., 2007), an observation that initially seemed paradoxical (Jortner et al., 2007). Finally, with thresholds so high, it is not surprising that the chances of “accidental” spiking are very small, and that KC spontaneous-firing rates are extremely low.

The design principles described here thus lead to reliable, specific—sparse as well as selective—representations of random olfactory-percepts in the mushroom body, and form a simple way to make a sparse code spontaneously emerge, with no need for a “guiding hand” such as learning or predetermined connections.

The total number of KCs, their fraction activated per external state, the levels of noise, and the cost function of classification errors will together determine how state-space is tiled—or how many odors can be reliably encoded by the mushroom bodies (and also, how many KCs are needed to encode a certain number of odors). A meaningful estimate is beyond the scope of this work, as a critical parameter—how distant KC representations must be from each other within noise constraints—is unknown.

Directly from the above principles emerges a simple recipe for designing networks with optimal separation of representations and arbitrarily specific responses. If two neuronal populations have feed-forward connectivity with probability ½, *N* source-neurons firing *p* spikes per characteristic time, and target neuron firing threshold is equivalent to $f(z)=\frac{Np+z\cdot \sqrt{Np\left(2-p\right)}}{2}$ (Appendix A5), then each target neuron will respond to a known proportion of source-population states (the area under Gaussian tail 1 − *Q*(*z*)), and different neurons will respond to maximally different states. Adaptive gain control should be implemented to ensure *f* changes appropriately when *p* changes (Papadopoulou et al., 2011). At any given time, target neurons' aggregate inputs (momentary membrane-potentials) will on average differ by $\frac{N\cdot p}{2}$.

### Generalization vs. discrimination

An inherent dilemma when parsing sensory input is where to draw category-lines. Sometimes a stimulus must be recognized—i.e., grouped into an already-existing category—even if it has never been previously encountered in the exact same form. This allows recognition of sensory stimuli in the presence of noise, as well as grouping things together into meaningful categories (i.e., generalization), both of which are essential requirements for the brain to perform its tasks.

In other cases, stimuli which may be very close to each other need to be told apart. Discrimination is critical when selecting food, for example. An extreme case is when the system needs to decide that something is completely novel and merits a new category of its own.

It is important to recognize that these tasks—discrimination and generalization—contradict each other to some extent, yet sensory systems need to be able to do both, and sometimes on the very same stimulus: something smells like a fruit (generalization), but clearly does not smell like an apple, though (discrimination).

The model presented here provides some intuition on how this may happen. As shown in Figures 6C,D, the same network can perform both tasks: with the sigmoid-shaped relation between source- and target-separation, stimuli close to each other at the source-layer will be generalized by the population, whereas stimuli farther from each other will be discriminated. The boundary between discrimination and generalization is rather sharp; and its location is determined by the firing threshold, which can be adapted (Papadopoulou et al., 2011).

### Kenyon cells can serve as building blocks for meaningful (and plastic) representations at their targets

The basic question we began our journey with is how the brain creates specific, high-level, and eventually ecologically meaningful percepts. The antennal-lobe–mushroom-body transformation described above achieves a major step in this direction by separating representations and giving rise to specific and random responses. However, it remains to be discussed how these random response properties lead to ecologically relevant percepts, and how this fits into the mushroom body's widely accepted role in learning [reviewed in Heisenberg (1998)].

The distribution of connection strengths between PNs and KCs is rather narrow (Jortner et al., 2007); in addition PN–KC synapses show no short-term plasticity, such as homo- or hetero-synaptic facilitation or depression (Jortner et al., 2007). While these observations do not rule plasticity out altogether, they definitely do not support plasticity playing a key role at PN–KC synapses.

What happens at the transformation to the next relay? Dendritic trees of β-lobe neurons (one of the main classes of mushroom body outputs) are planar and oriented perpendicular to the KC-axon tract; this structure suggests that β-lobe neurons can integrate precisely timed neural activity over a potentially wide subpopulation of KCs (Li and Strausfeld, 1997; MacLeod et al., 1998). Cassenaer and Laurent (2007) showed that connectivity from KCs to β-lobe neurons is low (~2%), individual active synapses are relatively strong (1.58 ± 1.11 mV) and exhibit salient spike-timing dependent plasticity, which is sensitive even to single action-potentials.

It is thus attractive to imagine the transformation of information from the antennal-lobe to the mushroom body as happening via widespread, random (or partially random) and largely fixed connections—designed to spread neuronal information optimally and create discrete, specific and reliable representations of random features. This would prepare it for further computation in downstream areas, such as the β-lobe—where more complex ideas can then be constructed from these elementary building blocks, much like words and phrases are constructed from an alphabet (Barlow, 1972; Stryker, 1992). Hence as different KCs respond specifically to various and different chemicals (or classes of chemicals), proper wiring of connections and selection/tuning of their strengths can generate high-level, invariant and “meaningful” representations. For example, a hypothetical downstream neuron responding only to odors associated with locust foods could easily be constructed by connecting onto it only KCs firing in response to various 5- and 6-carbon chained alcohols, aldehydes, and esters which are common odorants in grassy plants (cheerfully nicknamed “green odors”; Hopkins and Young, 1990; Bernays and Chapman, 1994). Similarly, some downstream neurons can respond to odors indicating plant toxicity (for examples of such chemical cues see Cottee et al., 1988).

At this downstream stage, learning (i.e., tweaking of incoming synapses from KCs) can shape and tune these representations, molding them to the animal's specific surroundings. Locusts, as many other generalist animals, readily adapt their food-preferences to seasonal- and regional-variation of plants, their nutritional value and the animal's needs (see Cooper-Driver et al., 1977; Bernays et al., 1992; Bernays and Chapman, 1994), and learning plays an important role in this (Dukas and Bernays, 2000; Behmer et al., 2005). It is likely, that learning a different food-preference is accomplished by changes at KC–β-lobe synapses, based on positive- and/or negative-reinforcement signals—originating, for example, from the digestive system (Behmer et al., 1999) and relayed via neuromodulatory reward/punishment signals, as shown in a variety of insect species (Hammer and Menzel, 1995, 1998; Schwaerzel et al., 2003; Unoki et al., 2005).

Learning can thus sculpt and tune higher neuronal representations, bringing neurons downstream of the mushroom body to respond to “meaningful” stimuli; i.e., stimuli with ecological importance for the animal (Barlow, 1972, 1985). Wiring each of these β-lobe neurons further, to directly trigger a relevant motor-program (e.g., for eating, avoidance, escape, etc.) would close the loop from perception to action. This would result in a simple neural system which receives complex, high-dimensional and noisy input and produces reliable animal behavior in response to it—in other words, a simple brain that works—and where we are approaching a deeper mechanistic understanding of the process.

### Switching between coding schemes

A large body of work is focused on sparse codes, pointing out their many benefits (e.g., Barlow, 1972; Palm, 1980; Baum et al., 1988; Kanerva, 1988, 1993; Földiák, 1990, 2002). Sparse codes are attractively easy to read out, as few spikes from few neurons translate to meaning, eliminating the need to integrate over an entire population or over a long time (Földiák, 1990, 2002). Forming associations is easy, as learning has to act on few nodes; in the theoretical limit-case (one-neuron-per-percept) tuning a single synapse suffices to (asymmetrically) link two percepts (Palm, 1980; Baum et al., 1988; Kanerva, 1993). Complex, meaningful ideas can be constructed by wiring-together basic random percepts (see Section “Kenyon Cells Can Serve as Building Blocks for Meaningful (and Plastic) Representations at their Targets”). Finally, sparse codes are metabolically economical, although an energetic trade-off exists between firing few spikes and maintaining many cells (Levy and Baxter, 1996; Attwell and Laughlin, 2001). Sparse codes are thus attractive and economical substrates for computation.

On the other hand, sparse coding has several serious drawbacks. It is wasteful in hardware, as each neuron participates only in a small fraction of representations (each percept requires devoted neurons and representations rarely share the same cells). It is also extremely sensitive to neuronal damage, as losing neurons results in loss of precepts or memories (Földiák, 2002).

Sparse codes thus seem unlikely candidates for applications such as long-term memory storage, but they are very well suited for applications such as short-term memory formation and associative learning. I find it attractive to envision the brain as functioning by transitioning back and forth between sparse and distributed coding schemes across different regions, according to the computations needed (Baum et al., 1988; Földiák, 1990, 2002). The design principles emerging from the present study suggest a neural algorithm, or a recipe, of how such transition may be (biologically and algorithmically) accomplished.

### Regaining complexity: re-examining the model's initial assumptions

The model I presented here relies on several rather crude approximations and assumptions (set forth in Section “Model Assumptions”). I now re-examine them in light of experimental data, and wherever they deviate from biological reality, try to assess how the model's predictions are affected. In other words, it's time to make things complicated again.

My first assumption was using discrete time windows, during which PN-spiking is treated as binary (firing or not). The locust olfactory system operates with an internal 20-Hz clock imposed on it (Laurent and Davidowitz, 1994; Laurent and Naraghi, 1994; Perez-Orive et al., 2002, 2004); PNs rarely fire more than once per 50 ms cycle (Perez-Orive et al., 2002, 2004), with odor-evoked spikes confined to the 25 ms rising-phase of the local field-potential oscillation (Laurent and Davidowitz, 1994; Laurent et al., 1996; Wehr and Laurent, 1996). The assumption is thus justified, at least for odor conditions. During baseline the coherence of the PN population is much reduced (as reflected by local-field potentials), yet most PNs retain a 20-Hz oscillatory component, as spike-autocorrelations show (Jortner et al., 2007). On average, the minimal PN-inter-spike-interval during baseline is ~22 ms; so there are definitely sometimes two spikes per arbitrary 50-ms window, although rarely more than two (Jortner, 2009). This deviation from model assumptions could increase the number of EPSPs summing per time window in a KC during baseline, and reduce the number of inputs needed for threshold-crossing. Having said this, the proportion of spikes with inter-spike-intervals below 50 ms is small, and as PN–KC synapses show no homo-synaptic facilitation on these time scales (Jortner et al., 2007), biases resulting from two spikes per window are expected to be small and linear (at most) with spike number.

A second assumption was i.i.d. spiking across different PNs over the course of the integration time—in other words, that PN activation-patterns are entirely random. This assumption simplified calculations and allowed applying the CLT to the summation of inputs. In reality, however, not all antennal-lobe states are equally probable given that the animal operates in a natural olfactory environment; in fact each individual locust is likely to experience in its lifetime only a miniscule fraction of the enormous number of possible PN activation patterns. Furthermore, during odor presentation PNs are affected by common excitatory input from ORNs and common inhibitory input from local interneurons, making randomness and mutual independence even less likely. In a nutshell, caveats of dependences and correlations between PNs may bias my analyses.

Several points support the model's conclusions despite this potential bias. First, simultaneous recordings show that spikes from different PNs are not correlated over short time scales at baseline (Jortner et al., 2007); this does not establish mutual independence but takes a step in that direction. Second, no direct synaptic connections were ever found between PNs (Jortner and Laurent, in preparation), which eliminates causality from contributing to statistical dependence. Finally, the classical CLT was extended for cases with dependences between variables (Bernstein, 1922, 1927), yielding modern versions of the theorem which hold under various mutual dependences and correlations (e.g., French and Wilson, 1978; Wilson, 1981; Reichert and Schilling, 1985; Pinske et al., 2007). The deviation from i.i.d. firing statistics needs, however, to be borne in mind.

As a third assumption, all PN–KC synaptic connections were treated as equal in strength. Experiments show PN–KC-EPSP amplitudes are distributed narrowly, but they are by no means uniform (86 ± 44 μV, with half of them within 60–110 μV; Jortner et al., 2007). Can ignoring the weights' distribution be justified? All calculations throughout this study were based on summation of rows of the connectivity matrix $\overset{↔}{W}$. If the number of summed elements *n* is large enough, the CLT justifies treating them as uniform (assuming i.i.d. between connections and finite variance, which is reasonable). This holds for “large enough” *n*, but is the length of a connectivity vector, or the number of EPSPs summating in a target neuron “large enough”? While the CLT strictly applies only when *n* approaches infinity, it in fact converges to Normality very fast as *n* starts to increase, then slows down asymptotically (established by the Berry–Esseen theorem: the difference between any CDF with finite variance and the Normal CDF decreases as $1/\sqrt{n}$; Feller, 1972). The number of summated connections in the model is on the order of *N* · *c* for connection vectors, and on the order of *N* · *c* · *p* for aggregate inputs—so for large *N* (800, in our case) the assumption is justified for all but very small values of *c* or of *c* · *p* (with locust parameters, *N* · *c* lies within the hundreds, and *N* · *c* · *p* is on the order of tens).

The last point has also been addressed directly with simulations in which sets of EPSPs from the experimental amplitude distribution were randomly drawn and summed (Jortner et al., 2007); for numbers ≥50, a Gaussian hypothesis for the sum could no longer be rejected, and differences between the actual sum and its estimate assuming uniformity were minute (Jortner et al., 2007, Supplementary Material). In conclusion, the model results are only minimally biased by the assumption of uniform connection strengths because the numbers are sufficiently high; this must be reexamined, however, if applying this framework to different systems where parameter values may be lower.

Fourth and last, PN–KC connectivity was treated as random. Previous work showed no obvious pattern in the pairs tested positive for connections; in fact, most KCs tested simultaneously with several PNs were found to be connected to about half of them (Jortner et al., 2007). Anatomically, the mushroom body calyx shows no simple patterning (e.g., layered- or columnar-organization) for either PN axons or KC dendrites (Farivar, 2005). Thus, no data has suggested patterning in the connectivity matrix. This having been said, it is very difficult (in fact, impossible) to establish true randomness in experimental data, and some patterns may have evaded my analysis. Even in such case, however, due to the huge number of combinations $\left(\left[\begin{array}{l} 800\\ 400 \end{array}\right]\approx 10^{240}\right)$ suggested by the data, any component of randomness in the connectivity matrix would still yield a combinatorial explosion of wiring possibilities—so very dramatic connection biases would be required to alter the conclusions of this study.

### Related models and alternative designs

In this study I took the gross structure of the locust olfactory system as starting point and basis for exploration; I did not explore all possible architectures or parameters, and by no means claim to offer the only solution for constructing specific representations from noisy input. A number of theoretical studies have tackled similar problems using different approaches, and have come up with a variety of designs. Here I briefly survey some of these models and their key properties, comparing and contrasting them with mine.

One example is Kanerva's (1988) Sparse Distributed Memory model. Its central idea—that memories can be represented as binary vectors in a high-dimensional space—stems from a key property of such spaces: points in them tend to differ from each other—and from most of the remaining space—along many dimensions. With this inherent sparseness in mind, a hyper-sphere is drawn around each point of interest (memory), and the memory is activated whenever an input vector falls within the hyper-sphere's boundaries; this grants the model noise-tolerance and flexibility more characteristic of brains than of most computers. As different hyper-spheres may partially overlap, input vectors often activate multiple memories. While performance depends on dimensionality, number of memories stored and activation radii, the model's main results are the feasibility and robustness of sparse-distributed storage and retrieval.

The Sparse Distributed Memory model is fully connected, meaning that when classifying an input, its values along all dimensions are taken into account; zeros as well as ones. This conveys the model its robustness, capacity, and noise tolerance. The threshold (the radius of the hyper-sphere) can be adapted if needed: for example, to ensure that state-space is tiled, or that each output responds with a particular probability. The high-dimensional space is thus filled with many partially overlapping hyper-spheres of the same dimension as the space; each represents one memory and its noise-tolerant envelope.

At another end of the spectrum of connectivity values is Jaeckel's (1989) Selected-Coordinate Design. In this model inputs also reside in a binary, high-dimensional space, yet each output samples just a handful of inputs (10 of 1000; corresponding to connection probability 0.01). For a memory to be activated, all of its sampled inputs (or selected coordinated) must take particular binary values; the rest of the inputs do not matter. Jaeckel's model thus attains its noise tolerance via invariance to most of the input's dimensions: it only takes into account 10 and ignores the rest. The threshold is thus fixed, and is equal to the number of selected coordinates (they all need to be active). In the Selected-Coordinate Design the high-dimensional space is thus inhabited by subspaces of lower dimensionality, each corresponding to a memory. To think in three dimensions, if input space were a cube, memories would be faces (squares) of this cube.

To compare my model with these, it is useful to speak a common language. In the locust, input space has 800 dimensions (one for each PN), so it is also high-dimensional and binary (as I treat each PN as spiking or not within each time window); PN–KC connectivity vectors correspond to points of interest in this space. The interesting properties of my model rely precisely on the inherent sparseness of high-dimensional binary spaces as formulated by Kanerva: PN–KC connectivity vectors populate a space so vast (containing roughly 10^240^ potential points) that the actual 5 × 10^4^ points realized tend to populate it extremely sparsely, each sitting on average very far from all others.

What does the portion of space which KCs respond to look like in my model, and how does their threshold affect it? In Kanerva's model each KC samples all the dimensions and is rather tolerant to errors in any of them (via the threshold); in Jaeckel's model it samples only very few dimensions, but is very strict about perfectly matching these. For comparison, in my model each KC samples half of the dimensions (corresponding to *c* = ½) and is invariant to the rest. This means that in 800-dimensional space, around half of the dimensions—those PNs which the KC is connected to—are treated as spherical, with a threshold, and the others are ignored, thus treated as cubical. The receptive range of a KC will thus be an 800-dimensional hyper-cylinder: spherical along some dimensions and invariant to the others. Adapting the threshold will only affect the spherical dimensions: the larger the radius, the lower the threshold, and thus the more states of the PN population activate the KC. The model suggested here thus combines the high-dimensionality and dense connectivity of Sparse Distributed Memory, the invariance to non-connected inputs from the Selected-Coordinate Design, and elements of noise-tolerance from both.

### Where else may these principles apply?

Neuronal specificity and sparse coding are widespread phenomena; relevant way beyond olfaction or sensory systems. The design principles discussed here are general in nature, relying on general assumptions and independent of particulars of the system. It is attractive to hypothesize that they may apply in a variety of other interconnected systems. While detailed data on network architecture—especially connection probabilities—is unfortunately still scarce for most biological networks, I point out several candidates which merit comparison.

What happens in other olfactory systems? In *Drosophila*, KCs are concentration invariant and much more specific than PNs (Turner et al., 2008; Honegger et al., 2011). KCs each seem to receive connections from around 10 PNs (corresponding to connectivity of ~5%), and have high firing thresholds (Turner et al., 2008). Differences in design and coding between locusts and flies may relate to their ecology: fruit flies occupy highly specialized ecological niches whereas locusts are generalist feeders. KC numbers also differ greatly across these species (50,000 in locust vs. 2500 in *Drosophila*); this could merely reflect size constraints, but may also relate to the extent of odor space the mushroom body needs to tile, or to the resolution required at different regions of the space.

Mammalian pyriform (olfactory) cortex shows similarities with the mushroom body: pyriform pyramidal neurons respond to odors with few spikes locked to respiratory oscillations and have low baseline firing rates (Poo and Isaacson, 2009). Pyriform cortex shows no evidence for spatial organization by odor tuning (Illig and Haberly, 2003; Rennaker et al., 2007; Stettler and Axel, 2009), and axons from individual mitral cells—its input neurons, analogous to insect-PNs—project onto it diffusely, without apparent spatial preference (Friedrich, 2011; Ghosh et al., 2011; Miyamichi et al., 2011; Sosulski et al., 2011). Connection probabilities between mitral cells and pyriform pyramidal cells are unknown; however, as these synapses are strong, coincident input from just a few may suffice to elicit spiking (Franks and Isaacson, 2006). This implies—albeit indirectly—that connection probabilities from second- to third-order neurons in rodents may be lower than in the locust. The level of sparseness of pyriform pyramidal neurons is 3–15%—also considerably lower than in locust KCs (Poo and Isaacson, 2009; Stettler and Axel, 2009; Isaacson, 2010). It remains to be seen how the various network parameters work in concert to yield coding solutions in this system.

One system often treated as a benchmark for decorrelation of representations is the cerebellum. Theoretical work by Marr (1969) and Kanerva (1988) suggests that the transformation from mossy fibers onto cerebellar granule cells is designed to decorrelate input representations and reduces the number of nodes learning would have to act on; operations precisely suited for a neural architecture such as described here. Measurements, however, indicate that convergence ratios of mossy fibers onto granule cells are much lower (Chadderton et al., 2004); the architecture described here may thus not apply to those neurons.

A fascinating candidate for comparison is the mammalian hippocampus. The ability to build meaningful representation from discrete random percepts makes sparse codes attractive for memory formation (Palm, 1980; Baum et al., 1988; Kanerva, 1988, 1993). This is a well-established role of both hippocampus (Scoville and Milner, 1957; Squire, 1992; Tulving and Markowitch, 1998) and mushroom body [reviewed in Heisenberg (1998)], and the analogy between the two has been previously drawn (Strausfeld et al., 1998). Indeed, similarly to KCs, some hippocampal neurons use extremely sparse codes: spiking specifically and reliably in response to complex, high-level stimuli and very rarely at baseline (Kreiman et al., 2000; Barnes et al., 2003; Quian Quiroga et al., 2005), with a majority silent at any given time (Thompson and Best, 1989). Topologically, hippocampus is largely feed-forward (Andersen et al., 1971; O'Reilly and McClelland, 1994; Andersen et al., 2000), and while its cytoarchitecture is extensively studied with classical anatomical techniques (e.g., Amaral and Witter, 1989; Patton and McNaughton, 1995), quantitative functional connectivity-data at single-cell resolution is just emerging (e.g., Brivanlou et al., 2004).

O'Reilly and McClelland (1994) provide in-depth theoretical analysis of hippocampal circuitry. They modeled feed-forward components of the circuit (entorhinal cortex, dentate gyrus, and CA3), exploring the effects of network parameters on pattern-separation and pattern-completion. Testing three values of feed-forward connectivity (equivalent to connection probability ~0.0001, 0.02, and 0.1), they indeed find that contrary to their intuition, the lowest connectivity value—which they had presumed to outperform the higher ones in pattern-separation—actually performed worse. They found performance similar between the higher values, suggesting a diminishing-returns effect; they did not, however, test higher connectivity-values approaching ~0.5. It would be tempting to test whether architecture within or among some hippocampal sub-regions (for example CA3–CA1) may follow similar design to the locust PN–KC circuitry, to maximize input separation as a basis for memory formation.

As the design discussed largely relies on random connectivity, it is not inherently suitable for circuits where the input's spatial relations must be retained, such as early visual- or auditory-areas. It may, however, apply well locally within spatially dependent modules, such as cortical columns (Mountcastle, 1997), or in higher processing areas, where representations become object-based and spatially invariant—such as infero-temporal cortex (Gross et al., 1972; Perrett et al., 1982; Fujita et al., 1992; Tanaka, 1996, 2003).

Optimal input-space separation may be useful even when sparse coding is not the goal: the targets' firing threshold determines response probability; if it is low, neurons will respond broadly. For example, connectivity ½ and a low firing threshold can generate distributed representations from sparse ones.

Core mechanisms elucidated here may still apply even with connectivity somewhat removed from the optimum: large enough cell-numbers, intermediate connectivity and some inherent randomness lead to a combinatorial explosion of wiring possibilities. This in turn naturally results in input spaces which are (by virtue of their mere size) extremely sparsely populated. A central message of this study is that to attain efficient input-spread, a suitable source–target connectivity regime is neither very dense, nor very sparse, but rather within an intermediate range.

## Concluding remarks: origins of neuronal specificity and the parsing of the olfactory world

Neuronal specificity and sparse neural coding have continuously attracted attention over several decades of brain research (e.g., Attneave, 1954; Marr, 1969, 1970; Willshaw and Longuet-Higgins, 1970; Barlow, 1972; Palm, 1980; Baum et al., 1988; Kanerva, 1988; Tsodyks and Feigel'Man, 1988; Perez-Vicente and Amit, 1989; Földiák, 1990; Rolls and Tovee, 1995; Vinje and Gallant, 2000; Willmore and Tolhurst, 2001; Simoncelli and Olshausen, 2001; Hahnloser et al., 2002; Laurent, 2002; Perez-Orive et al., 2002; Garcia-Sanchez and Huerta, 2003; DeWeese et al., 2003; Olshausen and Field, 2004; Huerta et al., 2004; Quian Quiroga et al., 2005; Jortner et al., 2007). One reason may be that they highlight a truly fundamental property of the brain: the ability to parse the surroundings and to extract meaning from them. Indeed, it seems that once a network of neurons can—through integration of external sensory inputs and a series of computations—bring single target-cells to respond differentially and reliably to particular objects, combinations of features or classes of stimuli, a significant part of the way towards performing the brain's tasks has already been made. A set of such “meaningfully responding” cells constitutes the very internal model of the world in the organism's brain—molded to its ecologically dictated requirements and reflecting the world as the animal views it. For example, characterization of a cell ensemble which represents a complex percept, such as an *Apple*, puts a handle on what thinking of an *Apple* is (Barlow, 1972); and strengthening a set of connections between this cell ensemble and another representing the concept of *Cake* creates both an associative link, and a higher, more complex idea. Mechanistic insights into how such representations come into being can open an intimate window onto the brain's subjective world-view and what forms it.

The system I have analyzed here does not yet offer direct access to this level of meaningful representations, but it does highlight the principles on the basis of which they can emerge. The principles along which the olfactory circuitry between the antennal-lobe and mushroom body is designed in the locust are an increase in dimensionality between source- and target-populations, feed-forward connectivity with probability of ½, maximizing separation between representations; and a high and adaptive firing threshold. This leads to specific, reliable, and sparse representations of random olfactory percepts in the mushroom body. Specificity is explained by a high enough threshold, only crossed when the KC encounters an appropriate input vector (from a set of vectors which lie within a particular radius of Hamming distances from an “ideal” central vector); very different from vectors which drive other KCs. Reliability results from the combination of strong convergence of PNs onto KCs (400:1) and cycle-by-cycle adjustment of the KC firing threshold (Papadopoulou et al., 2011).

In the following relay subsets of KCs are sampled by extrinsic β-lobe cells through sparse and strong synapses which are highly plastic (Cassenaer and Laurent, 2007); this can be used to build and learn meaningful representations for β-lobe neurons, constructed from the sparse discrete percepts randomly assigned to KCs (Barlow, 1972; Földiák, 2002). Cells responding to “meaningful” stimuli (for example, plants with high protein-content, or toxic plants) can directly activate motor programs—causing the insect to respond to the stimulus with an appropriate behavior (for example foraging or avoidance, respectively).

The locust olfactory circuitry emerges from this study as general-purpose machinery for information processing: a neural module which receives highly distributed and noisy inputs, spreads them maximally, and creates from them an arbitrarily sparse and selective set of representations—to be used as a substrate for learning, memory formation, categorization/generalization, triggering behavioral programs, and potentially a variety of other computations. These principles are suited to process any input where spatial relations need not be conserved, as they depend only to a limited extent on the nature of the signals to be processed. These mechanisms are therefore potentially of broad applicability and interest; where else they may apply remains to be seen.

### Conflict of interest statement

The author declares that the research was conducted in the absence of any commercial or financial relationships that could be construed as a potential conflict of interest.

## Appendix (Jortner, 2012)

### A1. Hamming distance between PN–KC connectivity vectors—full derivation

Let $\vec{U},\vec{V}$ be two arbitrary connectivity vectors, or rows of the connectivity matrix $\overset{↔}{W}$ (where 1 denotes connection, with probability *c*, and 0 none, with probability 1 − *c*) between the populations $\vec{S}$ and $\vec{K}$. The Hamming distance, $H(\vec{U},\vec{V})$, counts the number of bits different across the two vectors. I derive ${\langle H\left(\vec{U},\vec{V}\right)\rangle }_{U,V}$, the average Hamming distance between $\vec{U},\vec{V}$ over all their possible values:

$$\begin{array}{l} {\langle H\left(\vec{U},\;\vec{V}\right)\rangle }_{U,\;V}={\langle \sum_{i=1}^{N}{\left(U_{i}-V_{i}\right)}^{2}\rangle }_{U,\;V}\\ \;={\langle \sum_{i=1}^{N}\left(U_{i}^{2}-2U_{i}V_{i}+V_{i}^{2}\right)\rangle }_{U,\;V} \end{array}$$

as the average of a sum is the sum of the averages,

$$\begin{array}{l} ={\langle \sum_{i=1}^{N}U_{i}^{2}\rangle }_{U}-{\langle \sum_{i=1}^{N}2U_{i}V_{i}\rangle }_{U,\;V}+{\langle \sum_{i=1}^{N}V_{i}^{2}\rangle }_{V}\\ =\sum_{i=1}^{N}{\langle U_{i}^{2}\rangle }_{U}-2\sum_{i=1}^{N}{\langle U_{i}V_{i}\rangle }_{U,\;V}+\sum_{i=1}^{N}{\langle V_{i}^{2}\rangle }_{V} \end{array}$$

Finishing the calculation now requires the expected values of the expressions *U*^2^_*i*_, *V*^2^_*i*_, *U*_*i*_ *V*_*i*_. The following table gives all possible values of *U*_*i*_, *U*^2^_*i*_ and their respective probabilities:

**Table:**

***U*_*i*_**	***U*^2^_*i*_**	**Probability**
1	1	*c*
0	0	(1 − *c*)

So the expected value of 〈 *U*^2^_*i*_〉_*U*_ = 1 · *c* + 0 · (1 − *c*) = *c*; the same holds for 〈*V*^2^_*i*_〉_*V*_.

Here are all possible values of *U*_*i*_, *V*_*i*_, *U*_*i*_ *V*_*i*_ and their respective probabilities:

**Table:**

***U*_*i*_**	***V*_*i*_**	***U*_*i*_*V*_*i*_**	**Probability**
1	1	1	*c*^2^
1	0	0	*c* · (1 − *c*)
0	1	0	(1 − *c*) · *c*
0	0	0	(1 − *c*)^2^

So the expected value is

$${\langle V_{i}U_{i}\rangle }_{U,\;V}=1\;\cdot \;c^{2}+0\;\cdot \;\left(c\;\cdot \;(1-c)+(1-c)\;\cdot \;c+{\left(1-c\right)}^{2}\right)=c^{2}$$

Finishing the calculation:

$${\langle H(\vec{U},\;\vec{V})\rangle }_{U,\;V}=N\;\cdot \;c-2N\;\cdot \;c^{2}+N\;\cdot \;c=2N\;\cdot \;c\;\cdot \;(1-c)$$

### A2. Variance of input to a KC (Λ)—full derivation

I substitute the mean input to a KC by Ψ, which was already calculated (Section “Model Results II: Neuronal Activity and Properties of Input to KCs”):

$$\begin{array}{l} \Lambda \equiv {\langle \text{var}(k_{i})\rangle }_{i}={\langle {\langle {\left(k_{i}-{\langle k_{i}\rangle }_{\vec{S}}\right)}^{2}\rangle }_{\vec{S}}\rangle }_{i}={\langle {\langle {\left(k_{i}-\Psi \right)}^{2}\rangle }_{\vec{S}}\rangle }_{i}\\ \;={\langle {\langle {\left(\sum_{j=1}^{N}S_{j}W_{ij}-\Psi \right)}^{2}\rangle }_{\vec{S}}\rangle }_{i}\\ \;={\langle {\langle {\left(\sum_{j=1}^{N}S_{j}W_{ij}\right)}^{2}-2\Psi \sum_{j=1}^{N}S_{j}W_{ij}+\Psi^{2}\rangle }_{\vec{S}}\rangle }_{i}\\ \;={\langle {\langle \sum_{j=1}^{N}\sum_{k=1}^{N}S_{j}S_{k}W_{ij}W_{ik}-2\Psi \sum_{j=1}^{N}S_{j}W_{ij}+\Psi^{2}\rangle }_{\vec{S}}\rangle }_{i}\\ \;={\langle \sum_{j=1}^{N}\sum_{k=1}^{N}{\langle S_{j}S_{k}\rangle }_{\vec{S}}W_{ij}W_{ik}-2\Psi \sum_{j=1}^{N}{\langle S_{j}\rangle }_{\vec{S}}W_{ij}+\Psi^{2}\rangle }_{i} \end{array}$$

Next, I'll separate the first-term into its non-diagonal (*i* ≠ *j*) and diagonal (*i* = *j*) components and treat them each separately:

$$\Lambda ={\langle \sum_{j=1}^{N}\sum_{k=1,\;j\neq k}^{N}{\langle S_{j}S_{k}\rangle }_{\vec{S}}W_{ij}W_{ik}+\sum_{j=1}^{N}{\langle S_{j}^{2}\rangle }_{\vec{S}}W_{ij}^{2}-\ldots -2\Psi \sum_{j=1}^{N}{\langle S_{j}\rangle }_{\vec{S}}W_{ij}+\Psi^{2}\rangle }_{i}$$

To calculate the expected values of the non-diagonal terms *S*_*i*_, *S*_*j*_, *S*_*i*_ *S*_*j*_, the table below provides all possible values and their respective probabilities:

**Table:**

***S*_*i*_**	***S*_*j*_**	***S*_*i*_*S*_*j*_**	**Probablity**
1	1	1	*p*^2^
1	0	0	*p* · (1 − *p*)
0	1	0	(1 − *p*) · *p*
0	0	0	(1 − *p*)^2^

So the expected value is

$${\langle S_{i}S_{j}\rangle }_{\vec{S},\;i\neq j}=1\;\cdot \;p^{2}+0\;\cdot \;\left(p\;\cdot \;(1-p)+(1-p)\;\cdot \;p+{\left(1-p\right)}^{2}\right)=p^{2}$$

For the diagonal terms: *S*_*j*_, *S*^2^_*j*_ and their respective probabilities:

**Table:**

***S*_*j*_**	***S*^2^_*j*_**	**Probability**
1	1	*p*
0	0	(1 − *p*)

The expected value of ${\langle S_{j}^{2}\rangle }_{\vec{S}}=1\cdot p+0\cdot (1-p)=p$, the same holds for ${\langle S_{j}\rangle }_{\vec{S}}=1\cdot p+0\cdot (1-p)=p$

Continuing the calculation:

$$\Lambda =p^{2}\;\cdot \;\sum_{j=1\;}^{N}\sum_{k=1,\;j\neq k}^{N}{\langle W_{ij}W_{ik}\rangle }_{i}+p\;\cdot \;\sum_{j=1}^{N}{\langle W_{ij}^{2}\rangle }_{i}-\ldots -2\Psi \;\cdot \;p\;\cdot \;\sum_{j=1}^{N}{\langle W_{ij}\rangle }_{i}+\Psi^{2}$$

All possible values for 〈*W*_*ij*_ *W*_*ik*_〉_*i*, *j* ≠ *k*_, 〈 *W*^2^_*ij*_〉_*i*, *j*_, 〈 *W*_*ij*_〉_*i*, *j*_ and their respective probabilities:

**Table:**

***W*_*ij*_**	***W*_*ik*_**	***W*_*ij*_*W*_*ik*_**	**Probability**
1	1	1	*c*^2^
1	0	0	*c* · (1 − *c*)
0	1	0	(1 − *c*) · *c*
0	0	0	(1 − *c*)^2^

So the expected value is

$${\langle W_{ij}W_{ik}\rangle }_{i,\;j\neq k}=1\;\cdot \;c^{2}+0\;\cdot \;(c\;\cdot \;(1-c)+(1-c)\;\cdot \;c+{\left(1-c\right)}^{2})=c^{2}$$

**Table:**

***W*_*ij*_**	***W*^2^_*ij*_**	**Probability**
1	1	*c*
0	0	(1 − *c*)

the expected value of 〈 *W*^2^_*ij*_〉_*i*, *j*_ = 1 · *c* + 0 · (*1* − *c*) = *c* and the same holds for 〈 *W*_*ij*_〉_*i*, *j*_ = 1 · *c* + 0 · (1 − *c*) = *c*

There are exactly (*N*^2^ − *N*) non-diagonal terms, and *N* diagonal terms, so

$$\Lambda =p^{2}\;\cdot \;\left(N^{2}-N\right)\;\cdot \;c^{2}+p\;\cdot \;N\;\cdot \;c-2\Psi \;\cdot \;p\;\cdot \;N\;\cdot \;c+\Psi^{2}$$

I will now substitute back Ψ = *N* · *p* · *c* (as shown in Section “Model Results II: Neuronal Activity and Properties of Input to KCs”), and get:

$$\begin{array}{l} \Lambda =\Psi^{2}-p^{2}\;\cdot \;N\;\cdot \;c^{2}+p\;\cdot \;N\;\cdot \;c-2\Psi^{2}+\Psi^{2}\\ \;=N\;\cdot \;p\;\cdot \;c\;\cdot \;(1-p\;\cdot \;c)=N\;\cdot \;p\;\cdot \;c\;\cdot \;(1-p\;\cdot \;c) \end{array}$$

### A3. Covariance of the inputs to two KCs—full derivation

To calculate the covariance between inputs (or between sub-threshold membrane potentials) of two KCs, I substitute the mean input to a KC by Ψ, which was already calculated (Section “Model Results II: Neuronal Activity and Properties of Input to KCs”).

$$\begin{array}{l} {\langle \text{cov}(k_{r},\;k_{t})\rangle }_{r\neq t}={\langle {\langle \left(k_{r}-{\langle k_{r}\rangle }_{\vec{S}}\right)\left(k_{t}-{\langle k_{t}\rangle }_{\vec{S}}\right)\rangle }_{\vec{S}}\rangle }_{r\neq t}\\ \;={\langle {\langle \left(\sum_{i=1}^{N}S_{i}W_{ri}-\Psi \right)\left(\sum_{j=1}^{N}S_{j}W_{tj}-\Psi \right)\rangle }_{\vec{S}}\rangle }_{r\neq t}\\ \;={\langle {\langle \sum_{i=1}^{N}\sum_{j=1}^{N}S_{i}S_{j}W_{ri}W_{tj}-\Psi \sum_{i=1}^{N}S_{i}W_{ri}-\ldots -\Psi \sum_{j=1}^{N}S_{j}W_{tj}+\Psi^{2}\rangle }_{\vec{S}}\rangle }_{r\neq t}\\ \;={\langle \sum_{i=1}^{N}\sum_{j=1}^{N}{\langle S_{i}S_{j}\rangle }_{\vec{S}}\;\cdot \;W_{ri}W_{tj}-\ldots -\Psi \sum_{i=1}^{N}{\langle S_{i}\rangle }_{\vec{S}}\;\cdot \;W_{ri}-\Psi \sum_{j=1}^{N}{\langle S_{j}\rangle }_{\vec{S}}\;\cdot \;W_{tj}+\Psi^{2}\rangle }_{r\neq t} \end{array}$$

separating the first-term into non-diagonal and diagonal components:

$${\langle \sum_{i=1}^{N}\sum_{j=1,\;i\neq j}^{N}{\langle S_{i}S_{j}\rangle }_{\vec{S}}\;\cdot \;W_{ri}W_{tj}+\sum_{i=1}^{N}{\langle S_{i}^{2}\rangle }_{\vec{S}}\;\cdot \;W_{ri}W_{ti}-\ldots -\Psi \sum_{i=1}^{N}{\langle S_{i}\rangle }_{\vec{S}}\;\cdot \;W_{ri}-\Psi \sum_{j=1}^{N}{\langle S_{j}\rangle }_{\vec{S}}\;\cdot \;W_{tj}+\Psi^{2}\rangle }_{r\neq t}$$

I calculate the expected values of the terms *S*_*i*_ *S*_*j*_, *S*^2^_*i*_, *S*_*i*_ exactly as in Appendix A2:

$${\langle p^{2}\;\cdot \;\sum_{i=1}^{N}\sum_{j=1,\;i\neq j}^{N}W_{ri}W_{tj}+p\;\cdot \;\sum_{i=1}^{N}W_{ri}W_{ti}-p\;\cdot \;\Psi \sum_{i=1}^{N}W_{ri}-\ldots -p\;\cdot \;\Psi \sum_{j=1}^{N}W_{tj}+\Psi^{2}\rangle }_{r\neq t}=p^{2}\;\cdot \;\sum_{i=1}^{N}\sum_{j=1,\;i\neq j}^{N}{\langle W_{ri}W_{tj}\rangle }_{r\neq t}+p\;\cdot \;\sum_{i=1}^{N}{\langle W_{ri}W_{ti}\rangle }_{r\neq t}-\ldots -p\;\cdot \;\Psi \sum_{i=1}^{N}{\langle W_{ri}\rangle }_{r\neq t}-p\;\cdot \;\Psi \sum_{j=1}^{N}{\langle W_{tj}\rangle }_{r\neq t}+\Psi^{2}$$

and because of the condition *r* ≠ *t*, the expected value of the term 〈 *W*_*ri*_ *W*_*tj*_ 〉_*r* ≠ *t*, *i* ≠ *j*_ is identical to that of 〈 *W*_*ri*_ *W*_*ti*_ 〉_*r* ≠ *t*_, both equal to *c*^2^. The rest of the terms are calculated exactly as in A2, so:

$$\begin{array}{l} p^{2}\;\cdot \;(N^{2}-N)\;\cdot \;c^{2}+p\;\cdot \;N\;\cdot \;c^{2}-p\;\cdot \;\Psi \;\cdot \;N\;\cdot \;c-p\;\cdot \;\Psi \;\cdot \;N\;\cdot \;c+\Psi^{2}=\Psi^{2}-p^{2}\;\cdot \;N\;\cdot \;c^{2}+p\;\cdot \;N\;\cdot \;c^{2}-\Psi^{2}-\Psi^{2}+\Psi^{2}\\ \;=N\;\cdot \;c^{2}\;\cdot \;p\;\cdot \;(1-p) \end{array}$$

### A4. Difference between two KC inputs (or sub-threshold membrane potentials)

Here I follow the exact same lines of reasoning as in Appendix A1–A3. First, I express the difference between KC inputs, second, I split it into its non-diagonal and diagonal components, and third, I average each class of terms separately:

$$\begin{array}{l} D(k_{r},\;k_{t})\equiv {\langle {\langle {\left(k_{r}-k_{t}\right)}^{2}\rangle }_{\vec{S}}\rangle }_{r\neq t}={\langle {\langle {\left(\sum_{i=1}^{N}S_{i}W_{ri}-\sum_{j=1}^{N}S_{j}W_{tj}\right)}^{2}\rangle }_{\vec{S}}\rangle }_{r\neq t}\\ \;={\langle {\langle \left(\sum_{i=1}^{N}S_{i}W_{ri}-\sum_{j=1}^{N}S_{j}W_{tj}\right)\;\cdot \;\left(\sum_{m=1}^{N}S_{m}W_{rm}-\sum_{n=1}^{N}S_{n}W_{tn}\right)\rangle }_{\vec{S}}\rangle }_{r\neq t}\\ \;={\langle {\langle \sum_{i=1}^{N}\sum_{m=1}^{N}S_{i}S_{m}W_{ri}W_{rm}\rangle }_{\vec{S}}\rangle }_{r\neq t}-{\langle {\langle \sum_{i=1}^{N}\sum_{n=1}^{N}S_{i}S_{n}W_{ri}W_{tn}\rangle }_{\vec{S}}\rangle }_{r\neq t}-\ldots -{\langle {\langle \sum_{j=1}^{N}\sum_{m=1}^{N}S_{j}S_{m}W_{tj}W_{rm}\rangle }_{\vec{S}}\rangle }_{r\neq t}+{\langle {\langle \sum_{j=1}^{N}\sum_{n=1}^{N}S_{j}S_{n}W_{tj}W_{tn}\rangle }_{\vec{S}}\rangle }_{r\neq t}\\ \;={\langle {\langle \sum_{i=1}^{N}\sum_{m=1,\;i\neq m}^{N}S_{i}S_{m}W_{ri}W_{rm}\rangle }_{\vec{S}}\rangle }_{r\neq t}+{\langle {\langle \sum_{i=1}^{N}S_{i}^{2}\;\cdot \;W_{ri}^{2}\rangle }_{\vec{S}}\rangle }_{r\neq t}-\ldots -{\langle {\langle \sum_{i=1}^{N}\sum_{n=1,\;i\neq n}^{N}S_{i}S_{n}W_{ri}W_{tn}\rangle }_{\vec{S}}\rangle }_{r\neq t}-{\langle {\langle \sum_{i=1}^{N}S_{i}^{2}W_{ri}W_{ti}\rangle }_{\vec{S}}\rangle }_{r\neq t}-\ldots -{\langle {\langle \sum_{j=1}^{N}\sum_{m=1,\;j\neq m}^{N}S_{j}S_{m}W_{tj}W_{rm}\rangle }_{\vec{S}}\rangle }_{r\neq t}-{\langle {\langle \sum_{j=1}^{N}S_{j}^{2}W_{tj}W_{rj}\rangle }_{\vec{S}}\rangle }_{r\neq t}+\ldots +{\langle {\langle \sum_{j=1}^{N}\sum_{n=1,\;j\neq n}^{N}S_{j}S_{n}W_{tj}W_{tn}\rangle }_{\vec{S}}\rangle }_{r\neq t}+{\langle {\langle \sum_{j=1}^{N}S_{j}^{2}W_{tj}^{2}\rangle }_{\vec{S}}\rangle }_{r\neq t}\\ \;=\sum_{i=1}^{N}\sum_{m=1,\;i\neq m}^{N}{\langle S_{i}S_{m}\rangle }_{\vec{S}}\;\cdot \;{\langle W_{ri}W_{rm}\rangle }_{r\neq t}+\sum_{i=1}^{N}{\langle S_{i}^{2}\rangle }_{\vec{S}}\;\cdot \;{\langle W_{ri}^{2}\rangle }_{r\neq t}-\ldots -\sum_{i=1}^{N}\sum_{n=1,\;i\neq n}^{N}{\langle S_{i}S_{n}\rangle }_{\vec{S}}\;\cdot \;{\langle W_{ri}W_{tn}\rangle }_{r\neq t}-\sum_{i=1}^{N}{\langle S_{i}^{2}\rangle }_{\vec{S}}\;\cdot \;{\langle W_{ri}W_{ti}\rangle }_{r\neq t}-\ldots -\sum_{j=1}^{N}\sum_{m=1,\;j\neq m}^{N}{\langle S_{j}S_{m}\rangle }_{\vec{S}}\;\cdot \;{\langle W_{tj}W_{rm}\rangle }_{r\neq t}-\sum_{j=1}^{N}{\langle S_{j}^{2}\rangle }_{\vec{S}}\;\cdot \;{\langle W_{tj}W_{rj}\rangle }_{r\neq t}+\ldots +\sum_{j=1}^{N}\sum_{n=1,\;j\neq n}^{N}{\langle S_{j}S_{n}\rangle }_{\vec{S}}\;\cdot \;{\langle W_{tj}W_{tn}\rangle }_{r\neq t}+\sum_{j=1}^{N}{\langle S_{j}^{2}\rangle }_{\vec{S}}\;\cdot \;{\langle W_{tj}^{2}\rangle }_{r\neq t}\\ \;=N(N-1)\;\cdot \;p^{2}\;\cdot \;c^{2}+N\;\cdot \;p\;\cdot \;c-N(N-1)\;\cdot \;p^{2}\;\cdot \;c^{2}-N\;\cdot \;p\;\cdot \;c^{2}-\ldots -N(N-1)\;\cdot \;p^{2}\;\cdot \;c^{2}-N\;\cdot \;p\;\cdot \;c^{2}+N(N-1)\;\cdot \;p^{2}\;\cdot \;c^{2}+N\;\cdot \;p\;\cdot \;c\\ \;=2N\;\cdot \;p\;\cdot \;c\;\cdot \;(1-c) \end{array}$$

### A5. Firing threshold when connectivity is 1/2

A threshold designed to be crossed for a particular fraction of states 1 − *Q*(*z*), where *Q* is the CDF of the standard Normal distribution, should be equal to the mean (Ψ) plus the appropriate times the standard deviation ($\sqrt{\Lambda }$).

Assuming *c*= ½, we get:

$$\begin{array}{l} f(z)=\Psi +z\sqrt{\Lambda }=N\;\cdot \;p\;\cdot \;c+z\sqrt{N\;\cdot \;p\;\cdot \;c\;\cdot \;(1-p\;\cdot \;c)}\\ \;c=\frac{1}{2}\Rightarrow f(z)=\frac{N\;\cdot \;p}{2}+z\sqrt{\frac{N\;\cdot \;p}{2}\left(1-\frac{p}{2}\right)}=\frac{N\;\cdot \;p+z\sqrt{N\;\cdot \;p\;\cdot \;\left(2-p\right)}}{2} \end{array}$$
